# Sulfation modification of dopamine in brain regulates aggregative behavior of animals

**DOI:** 10.1093/nsr/nwab163

**Published:** 2021-09-02

**Authors:** Bing Chen, Xiwen Tong, Xia Zhang, Wanying Gui, Guoming Ai, Lihua Huang, Ding Ding, Jiangxu Zhang, Le Kang

**Affiliations:** School of Life Science, Institutes of Life Science and Green Development, Hebei University, Baoding 071002, China; State Key Laboratory of Integrated Management of Pest Insects and Rodents, Institute of Zoology, Chinese Academy of Sciences, Beijing 100101, China; State Key Laboratory of Integrated Management of Pest Insects and Rodents, Institute of Zoology, Chinese Academy of Sciences, Beijing 100101, China; School of Life Sciences, South China Normal University, Guangzhou 510631, China; State Key Laboratory of Integrated Management of Pest Insects and Rodents, Institute of Zoology, Chinese Academy of Sciences, Beijing 100101, China; State Key Laboratory of Integrated Management of Pest Insects and Rodents, Institute of Zoology, Chinese Academy of Sciences, Beijing 100101, China; State Key Laboratory of Microbial Resources, Institute of Microbiology, Chinese Academy of Sciences, Beijing 100101, China; School of Life Sciences, South China Normal University, Guangzhou 510631, China; State Key Laboratory of Integrated Management of Pest Insects and Rodents, Institute of Zoology, Chinese Academy of Sciences, Beijing 100101, China; State Key Laboratory of Integrated Management of Pest Insects and Rodents, Institute of Zoology, Chinese Academy of Sciences, Beijing 100101, China; School of Life Science, Institutes of Life Science and Green Development, Hebei University, Baoding 071002, China; State Key Laboratory of Integrated Management of Pest Insects and Rodents, Institute of Zoology, Chinese Academy of Sciences, Beijing 100101, China

**Keywords:** behavioral plasticity, sulfation, dopamine, aggregation, migratory locust

## Abstract

Behavioral plasticity and the underlying neuronal plasticity represent a fundamental capacity of animals to cope with environmental stimuli. Behavioral plasticity is controlled by complex molecular networks that act under different layers of regulation. While various molecules have been found to be involved in the regulation of plastic behaviors across species, less is known about how organisms orchestrate the activity of these molecules as part of a coherent behavioral response to varying environments. Here we discover a mechanism for the regulation of animal behavioral plasticity involving molecular sulfation in the brain, a modification of substrate molecules by sulfotransferase (ST)-catalyzed addition of a sulfonate group (SO_3_) from an obligate donor, 3^′^-phosphoadenosine 5^′^-phosphosulfate (PAPS) to the substrates. We investigated aggregation behaviors of migratory locusts, which are well-known for extreme phase change plasticity triggered by population density. The processes of PAPS biosynthesis acted efficiently on induction of locust behavioral transition: Inhibition of PAPS synthesis solicited a behavioral shift from gregarious to solitarious states; external PAPS dosage, by contrast, promoted aggregation in solitarious locusts. Genetic or pharmacological intervention in the sulfation catalyzation resulted into pronounced solitarizing effects. Analysis of substrate-specific STs suggests a widespread involvement of sulfated neurotransmitters in the behavioral response. Dopamine in the brain was finally identified to be actively sulfate conjugated, and the sulfate conjugation enhanced the free DA-mediated behavioral aggregation. Similar results in *Caenorhabditis elegans* and mice indicate that sulfation may be involved more broadly in the modulation of animal aggregation. These findings reveal a general mechanism that effectively regulates animal social-like behavioral plasticity, possibly through sulfation-mediated modification of neural networks.

## INTRODUCTION

Behavioral plasticity of animals is a common phenomenon, in which an organism exhibits diverse behavioral traits in response to a range of environmental changes. Animals exhibit behavioral plasticity at multiple cognitive, sensory, social and motor levels. Behavioral plasticity is associated with neuronal plasticity, learning and memory, and is thus fundamental for animals [1]. Deficits in behavioral plasticity have been related to many behavioral abnormalities and neurological diseases [2].

Behavioral plasticity can be regulated by different molecules, such as neuropeptides, hormones and monoamines in both invertebrates and vertebrates [1,3–5]. In *Caenorhabditis elegans*, these molecules affect many aspects of behavior, such as food choice decision-making, aggregation and locomotion [1]. For example, the *npr-1* gene encoding a neuropeptide Y receptor regulates solitary or social locomotion behavior [6]. Another neuropeptide F and its receptor also modulate locomotor activity in the swarming of the migratory locust (Insecta: *Locusta migratoria*) [7]. Monoamine neurotransmitters such as tyramine, dopamine (DA) and 5-serotonin are also involved in aggregation behaviors in locust species [5,8,9]. Specifically, DA inside the brain plays a widespread role in the regulation of mobility, motivation, reward, attention and addiction. The DA biosynthetic metabolism and signaling pathway are crucial for regulation of olfactory attraction and mobility in the migratory locust [10,11]. However, research in neuronal regulation of locust behavioral aggregation has given various and sometimes conflicting mechanistic explanations in different species [8,9,12,13], such that a fundamental regulatory mechanism underpinning behavioral plasticity is still lacking. For example, DA plays a contrasting role in regulating the aggregation in the two notorious locust species, the migratory locust and the desert locust (*Schistocerca gregaria*) [8,14]. Another neurotransmitter, serotonin, also presented different behavioral roles in the two species [9,12]. While the molecules or their levels involved in the behavioral responses may have evolutionarily diverged among species, it remains elusive whether a mechanism coordinating the activity of these molecules and their networks could still be conserved.

The migratory locust is a well-established model of extreme phase change plasticity and displays two distinct behavioral phases, that is a cryptic solitarious phase (S) and a swarming gregarious phase (G) depending on population density. The locusts in S phase developing at low density are characterized by lethargy and conspecific repulsion. In contrast, the locusts in G phase develop at high density, and show high mobility and conspecific attraction [4]. Thus, the G locusts can aggregate and undergo collective migration. Molecular and neurological mechanisms regulating the phase change of the locust have been investigated from different aspects [4]. Several important biogenic amines, such as serotonin, dopamine and octopamine, have been attributed to modulation of locust aggregation behaviors [13]. cAMP-dependent protein kinase A, a signal transduction protein in aminergic signaling, is also involved in regulation of locust aggregation [15]. Noncoding RNAs involved in the metabolic synthesis of DA [16] or DA-induced adenylyl cyclase signaling [11] corroborate the central role of the DA signaling pathway in behavioral regulation.

**Figure 1.**
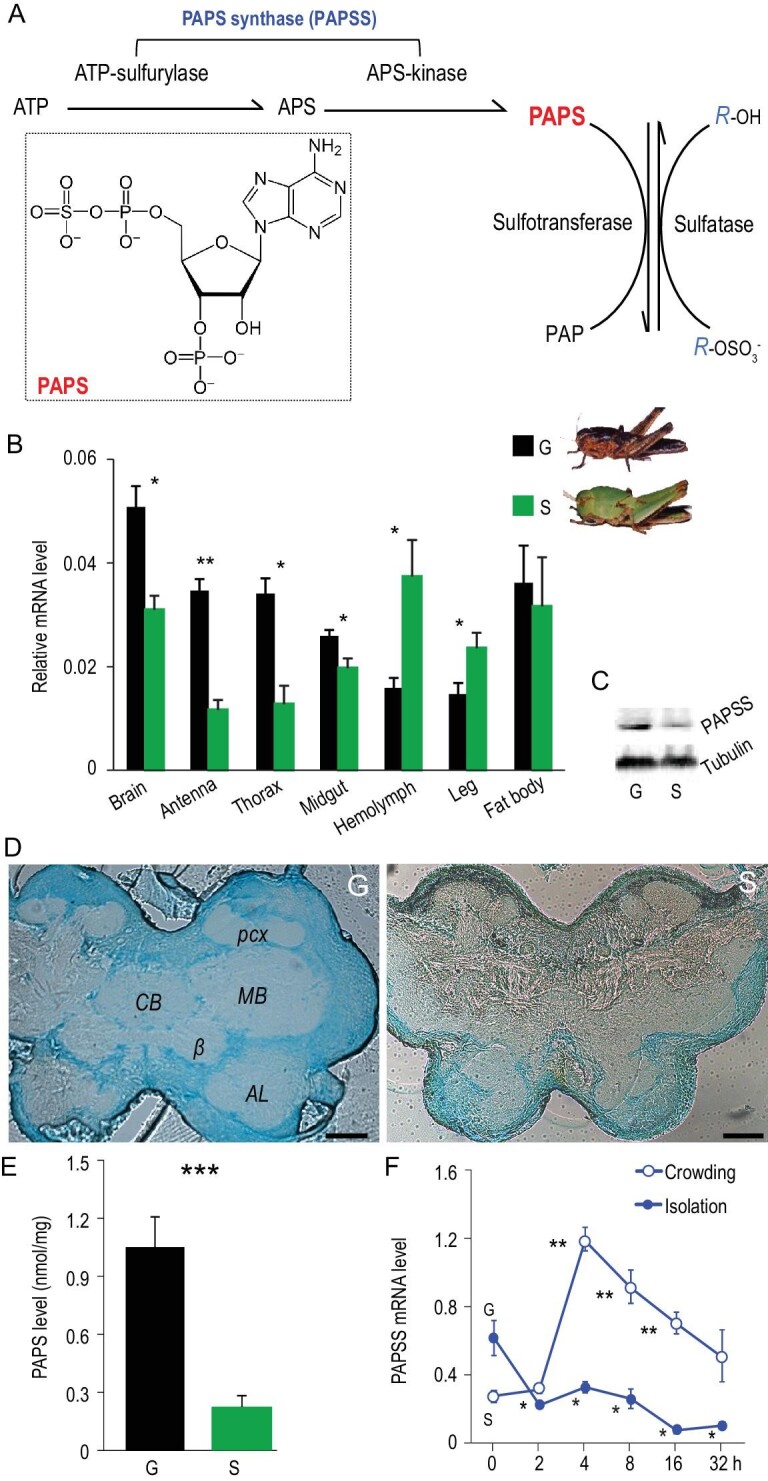
Locust brains are differentially sulfated in gregarious and solitarious phases. (A) Catalytic reactions in the formation of PAPS and sulfation with PAPS as the obligate sulfate donor. PAPS formation is catalyzed by the dual-function enzyme PAPS synthase (i.e. PAPSS). Sulfation is the sulfotransferase-catalyzed addition of a sulfuryl group, SO_3_, to the structure of a molecule (i.e. *R*). Desulfation is catalyzed by sulfatase. ATP, adenosine triphosphate; APS, adenosine-5'-phosphosulfate; PAPS, 3'-phosphoadenosine-5'-phosphosulfate (with its structure shown in the inset box); PAP, adenosine 3', 5'-diphosphate. (B) Tissue expression of *PAPSS* in the G and S phases of locust nymphs. G, gregarious locust; S, solitarious locust. The mRNA level was quantified by real-time PCR (qPCR) and normalized against that of the internal control gene *rp49*. Each tissue had five biological replicates of 10 nymphs. (C) PAPSS expression in brain was detected by western blot. Tubulin was used as internal control. (D) Sulfated molecules located in cell bodies were highly abundant in G relative to S. Sulfated glycan was detected by staining brain slices with 0.01% Alcian blue in 3% acetic acid, pH = 1.0. *pcx*, primary calyx; *MB*, mushroom body; *AL*, antennal lobe; *CB*, central body; *β*, β lobe. Scale bar, 100 μm. (E) G brains possessed 3.6-fold more PAPS than S (measured by HPLC-MS/MS, n = 6). (F) Brain expression of *PAPSS* increased during the time-course of nymph crowding, and reduced during isolation. Asterisks indicated significance differences between each time point with 0 hours (i.e. typical G or S locusts). mRNA levels were measured with five biological replicates of 10 brains. The data are shown as mean ± SEM. Student *t* test: ^*^, *P* < 0.05; ^*^^*^, *P* < 0.01; ^*^^*^^*^, P < 0.001.

Compared with their changes in expression levels, molecular modifications of the underlying molecules could be more important for fine tuning of behavioral plasticity. DNA or RNA modifications have been implicated in the locust phase changes [4,16]. Proteomic analysis of the G and S locusts showed that PAPS synthase (PAPSS) are among the highly differentially expressed proteins in the brain between the two phases [17]. PAPSS is the rate limiting enzyme in the process of sulfation, a reversible chemical modification of molecules including proteins, carbohydrates, hormones and bioamines [18,19]. Catalytic processes of sulfation involve two main steps, that is synthesis of the universal sulfate donor 3^′^-phosphoadenosine 5^′^-phosphosulfate (PAPS), by which a sulfate ion (SO_4_^2–^) is converted into PAPS, and the sulfonate moiety (SO_3_^2–^) in PAPS is introduced into the structure of a acceptor molecule [18] (Fig. 1A). Sulfation is critical for many biological processes, for example molecular recognition, signal transduction and hormone regulation [18]. Sulfation is involved in metabolism, development and physiology [20,21], as well as many pathophysiological processes [22,23]. A few pieces of evidence for involvement of sulfation in neuronal and behavioral regulation are emerging [24,25]. However, the role of brain-related sulfation modification in animal behavioral regulation remains largely unknown.

We hypothesized that sulfation may be involved in the modification of specific neuronal-related molecules that rewires their signaling networks governing behavioral plasticity elicited by population density in the locust. To test this hypothesis, we examined the role of the sulfation metabolic pathways, including the biosynthesis of PAPS and sulfation/desulfation, in the behavioral regulation of the locusts. Our study revealed a potent role of sulfation in regulation of behavioral aggregation not only in insect species but also in *C. elegans* and mice. Furthermore, we identified DA as a key sulfate-conjugated substrate in the brain and showed how DA is sulfated to promote the aggregation effects.

## RESULTS

### Elements of the sulfation pathway differ markedly between behavioral phases of the locust

We first examined whether locust brains differ in sulfation between the gregarious (G) and solitarious (S) phases. PAPSS is the PAPS synthase that catalyzes synthesis of PAPS in sulfation, and is bifunctional for ATP sulfurylase and adenosine 5^′^-phosphosulfate kinase [19] (Fig. 1A). Phylogenetic analysis showed that *PAPSS* are highly conserved in the nematode (*C. elegan*s), insects, mouse and human (Fig. S1A and B). Analysis of locust *PAPSS* expression in seven tissues of 4^th^-instar nymphs showed that *PAPSS* maintained a relatively high level in brains (Fig. 1B). Compared with S locusts, G locusts exhibited a much higher level of *PAPSS* in the brain (n = 5, *P* = 0.013 for mRNA level, Fig. 1B and C), which confirms previous results [17]. Sulfated molecules specifically stained by Alcian Blue presented in almost all the cell bodies that overlie the neuropils in the G brain. In contrast, sulfated substances in the brain of S locusts were much less abundant, and mainly distributed in part of the antennal lobe (Fig. 1D). A high performance liquid chromatography (HPLC) assay showed that G locusts also had a higher level of PAPS in the brain (*P* < 0.001, Fig. 1E). Furthermore, the expression level of *PAPSS* increased upon locust crowding and decreased immediately upon isolation, implying involvement of *PAPSS* in eliciting or maintaining swarming behavior in the locust (Fig. 1F). These results suggested a possible role of PAPS availability-limited sulfation in regulation of locust behavioral plasticity.

### Biosynthesis of the sulfate donor regulates locust behavioral transition

**Figure 2.**
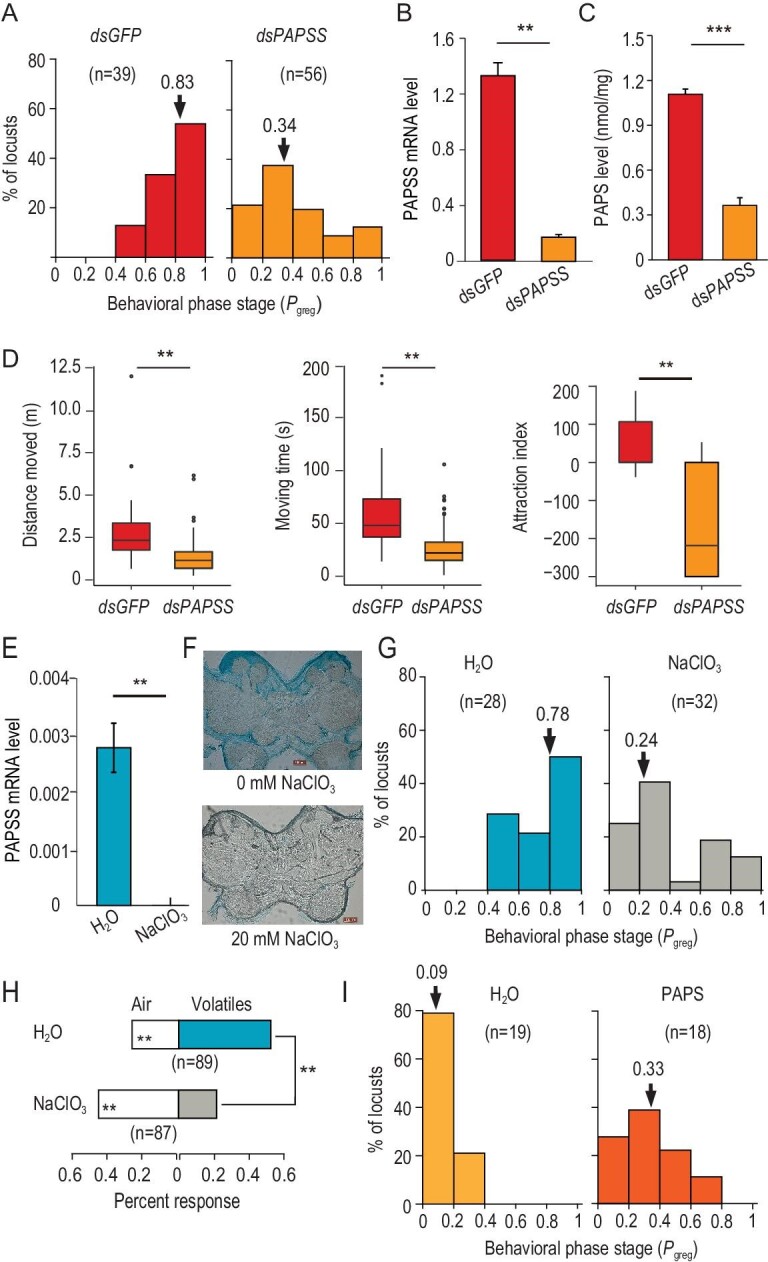
Biosynthesis of PAPS mediates locust behavioral transition between gregaria and solitaria. (A) *PAPSS* RNAi resulted in a behavioral transition from gregaria to solitaria at 24 hours after dsRNA injection. ds*PAPSS* represents *PAPSS* dsRNA injection. ds*GFP* represents *GFP* dsRNA. *P*_greg_ measured predisposition to swarming, 0, solitarious behavior; 1, gregarious behavior. Arrows indicate median *P*_greg_ values. n, number of individuals tested. (B and C) *PAPSS* RNAi reduced the mRNA level of *PAPSS* (n = 3, B) and synthesized PAPS (n = 6, C) in nymphal brain. (D) PAPSS knockdown reduced mobility and conspecific attraction of G nymphs. Mobility of nymphs was represented by the total distance moved and the duration of time of movement. Individual attraction was depicted by the attraction index. (E) Injection of PAPSS inhibitor, NaClO_3_, abolished *PAPSS* gene expression in brain (n = 3). (F) NaClO_3_ reduced expression of sulfated molecules in brain. Scale bar, 100 μm. (G) PAPSS inhibition by NaClO_3_ drove a behavioral shift from G to S phase of the locust. (H) NaClO_3_ injection caused a conversion from olfactory attraction to repulsion to conspecific odors. Dual-choice of locust nymphs to air or volatiles from 30 gregarious nymphs was examined in a Y-tube olfactometer. The asterisks inside the strip indicate the significance of differences in numbers in each arm. (I) PAPS induced locust aggregation at 1 hour after injection. The data are shown as mean ± SEM. ^*^^*^, *P* < 0.01; ^*^^*^^*^, *P* < 0.001.

We then explored whether the biosynthesis of the sulfate donor PAPS was involved in behavioral regulation of the locusts. We knocked down the expression of *PAPSS* by RNA interference (RNAi) in brains of G locusts and measured their behavioral states using an established automatic behavioral assay method [8,9,26]. Movement trajectories (over 300 s) of a locust exposed to stimulating sight and smell of other gregarious locusts in the behavioral arena were recorded using a video tracking system. Aggregation behavior was quantified by *P*_greg_, a summary statistic indicating gregarious state of a locust based on a binary logistic regression model that uses three behavioral metrics: the moving distance, the duration of movement and attraction index [8,9]. A *P*_greg_ of 1 represented fully gregarious behavior of an animal with long distance and duration of movement and high positive attraction index, whereas a *P*_greg_ of 0 indicated fully solitarious behavior of an animal with much less movement and lower or even negative attraction index. After injecting *PAPSS* double stranded RNA (dsRNA) for 24 hours, we observed a remarkable shift of injected G locusts towards solitarious behavioral traits (Mann-Whitney *U* test, *P* < 0.001; Fig. 2A). RNAi silencing of *PAPSS* expression also reduced the content of synthesized PAPS in the brain (Fig. 2B and C). The behavioral effects included reduced individual mobility in terms of the moving distance (Student *t* test, *P* < 0.001) and the duration of movement (Student *t* test, *P* < 0.001), as well as a shift from conspecific attraction to repulsion (Mann–Whitney *U* test, *P* = 0.003) (Fig. 2D). The solitarizing effects were still maintained at 48 hours after *PAPSS* dsRNA injection (Fig. S2A–E).

We further validated the regulatory role of PAPS synthesis using NaClO_3_, a potent inhibitor highly specific to PAPS synthase in various cells [27,28]. The inhibitory effects of NaClO_3_ on *PAPSS* expression were also apparent in the locust. NaClO_3_ injection abolished *PAPSS* expression in the locust brain (Fig. 2E), and removed sulfated substances in the neuropil of the middle brain such as the mushroom body, antennal lobe and central body (Fig. 2F). NaClO_3_ overdose did not result in apparent side effects on development, molting and survival of the locust. The chlorate injection caused a prominent behavioral shift from gregarious to solitarious traits, as indicated by a significant change in *P*_greg_ (Mann–Whitney *U* test, *U* = 262, *P* < 0.001, Fig. 2G). The behavioral changes of locusts encompassed a significant reduction of mobility in terms of total distance moved (*P* = 0.031, Fig. S2F), total duration of movement (*P* = 0.001, Fig. S2G), as well as a significant transition from conspecific attraction toward repulsion (*P* = 0.002, Fig. S2H). As olfactory cues contributed significantly to attraction behaviors [11,26], we subjected G locusts to a dual-choice between air and volatiles of other G locusts using a Y-tube olfactometer. The injection of chlorate caused a reduced preference of G locusts for conspecific volatiles (Fig. 2H). Thus, the synthesis of PAPS is involved in the olfactory sensation that contributes to conspecific attraction and aggregation.

The strict dependence of sulfation on PAPS availability as a sulfate donor implies a key regulatory role for supply of PAPS in behavioral regulation. Indeed, injection of PAPS in S locusts significantly promoted aggregation (*P* < 0.001; Fig. 2I and S3). We injected PAPS into the S locusts and monitored the behaviors at 1 hour and 4 hours after the injection. At 1 hour, the S locusts increased *P*_greg_ from 0.093 to 0.33, indicating a significant rapid change in phase-related behavior (*U* = 72, *P* < 0.001) (Fig. 2I). PAPS injection increased individuals’ mobility as shown by significantly elevated moving distance (*P* = 0.006) (Fig. S3A) and the duration of time of movement (*P *= 0.006) (Fig. S3B). The attraction index was also significantly altered after PAPS injection (*U* = 66, *P* < 0.001, Fig. S3C), indicating an increased preference for conspecific attraction. The gregarizing effects induced by PAPS injection still presented at 4 hours after the injection in terms of *P*_greg_ (*P* < 0.001, Fig. S3D), the total duration of movement (Fig. S3F) and attraction index (*P* < 0.001, Fig. S3G). Taken together, the biosynthesis and supply of PAPS are crucial for regulation of behavioral aggregation in the locusts.

### Cytosolic phenol sulfotransferases are responsible for the behavioral phase changes

**Figure 3.**
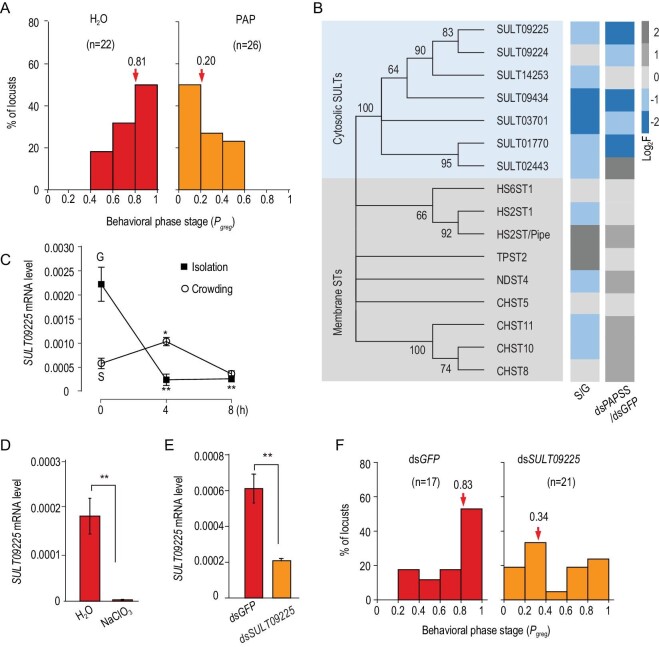
Phenol sulfotransferases are responsible for behavioral phase transition in the locust. (A) Injection of the sulfotransferase (ST) inhibitor PAP drove a completed behavioral shift from gregaria to solitaria. The behavior of nymphs was examined 1 hour after injection. Arrows indicate median *P*_greg_ values. (B) The expression levels of two types of ST genes revealed by phylogenetic analysis, i.e. cytosolic STs (i.e. SULTs) and Golgi membrane-bound STs. Heatmap indicates base-2 logarithmic ratio (R) of gene expression in RNAi (i.e. ds*PAPSS* versus ds*GFP*) or phase transition (i.e. S versus G) measured by qPCR. Expression was measured at 24 hours after RNAi. Three biological replicates of 10 brains were measured. (C) Brain expression of a cytosolic catecholamine SULT gene *SULT09225* during the time-course of nymph crowding and isolation. Asterisks indicate significance differences between each time point and 0 hours (i.e. typical G or S locusts). The mRNA level was normalized against that of the internal control gene *rp49*. Each treatment had three biological replicates of 10 brains. (D) NaClO_3_ injection abolished expression of *SULT09225* (n = 3). (E) *SULT09225* expression knocked down by dsRNA injection in brain (n = 3). (F) Knockdown of *SULT09225* caused a behavioral transition from gregaria to solitaria. The data were shown as mean ± SEM. ^*^, *P* < 0.05; ^*^^*^, *P* < 0.01; ^*^^*^^*^, *P* < 0.001.

We next explored whether the catalytic reaction of sulfation could mediate locust behavioral plasticity. The sulfation reaction is catalyzed by sulfotransferases (STs) to introduce a sulfate group of PAPS into the structure of a molecule (i.e. substrate). Adenosine 3^′^, 5^′^-diphosphate (PAP) is a potent competitive inhibitor of STs, and most effective for the transformation of DA and phenol substrates [18,29]. We examined the behavioral changes linked to pharmacological inhibition of the sulfation reactions. The PAP injection solicited a prominent and rapid behavioral shift from gregarious to solitarious traits, with a remarkable change of the median *P*_greg_ value from 0.2 to 0.8 at 1 hour (*P* < 0.001; Fig. 3A). The result suggested a potent role of STs in the behavioral regulation.

We next investigated which types of biomolecules were potentially sulfated: small compounds such as monoamines, or bigger biomolecules such as proteins. We decided to examine ST families because of their substrate specificity. We identified 16 STs in the locust genome whose transcripts were confirmed by PCR and sequencing. Phylogenetic analysis of the 16 locust STs showed that seven STs belonged to the clades of cytosolic ST (thereafter designated as SULTs), sulfating small molecules such as steroid hormones and catecholamines, and nine STs belonged to the Golgi-membrane bound STs, sulfating larger biomolecules including glycosaminoglycans and proteins [18,19,30] (Fig. 3B). Expression assays of these *SULT*s showed that 12 out of the 16 *SULT*s differentially expressed in the G and S locusts, and almost all cytosolic *SULT*s (six out of seven) were upregulated in G locusts. *PAPSS* knockdown resulted in downregulation of five cytosolic *SULT*s, but no downregulation of membrane *SULT*s at 24 hours upon the RNAi (Fig. 3B). The results implied that cytosolic SULTs were actively involved in the sulfation in response to changes of locust density.

We further analyzed the expression of these cytosolic *SULT*s during time-course isolation and crowding of locusts. *SULT09225* exhibited a dramatic decrease of mRNA expression levels in response to isolation and an increase in response to crowding (Fig. 3C). *SULT03701* demonstrated no response. The other cytosolic *SULT*s also presented expression changes, but at certain time points of the isolation or crowding courses (Fig. S4). Alignment analysis showed that SULT09225 is highly conserved with human cytosolic SULTs at many sequences including Region I and Region IV that are important for PAPS binding [30] (Fig. S5). Based on sequence homology, *SULT09225* possibly encodes an ortholog of human SULT that preferentially conjugates catecholamine neurotransmitters [18,19] (Fig. S5). NaClO_3_ injection almost abolished expression of *SULT09225* in locust brain (*P* = 0.009; Fig. 3D), confirming the involvement of *SULT09225* in the sulfation process.

We then tested whether *SULT09225* is involved in regulation of the aggregative behaviors of the locust. We knocked down *SULT09225* expression in the brain by RNAi (*P* = 0.007; Fig. 3E). The knockdown resulted in a significant behavioral shift of G locusts toward solitarious traits (*P* = 0.021; Fig. 3F). Taken together, these results suggested that the *SULT*s conjugating phenolic substrates such as catecholamines are involved in the behavioral regulation.

### DA in brain is an active substrate sulfated to promote behavioral aggregation

**Figure 4.**
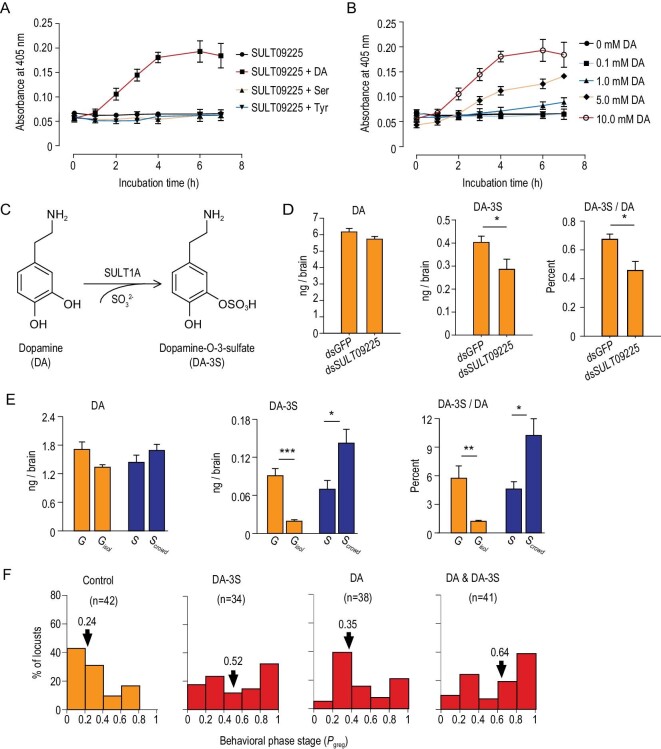
Dopamine in brain is sulfated to promote behavioral phase transition in the locust. (A) Recombinant expressed SULT09225 catalyzed dopamine (DA) but not 5-serotonin (Ser) and tyramine (Tyr). The colorimetric assay on at least three independent experiments in triplicate was performed. Concentrations of 10 mM DA, 5 mM Ser and 10 mM Tyr were used. (B) Concentration response for sulfation of DA by SULT09225. (C) The sulfation of dopamine (DA) into dopamine-*O*-3-sulfate (DA-3S) catalyzed by a catecholamine SULT enzyme. (D) *SULT09225* RNAi reduced the content of DA-3S and the proportion of DA-3S relative to DA in brain. The level of DA and DA-3S was measured by LC-MS/MS in at least five replicates of 20 nymphal brains. (E) The content of DA-3S and the proportion of DA-3S relative to DA was greatly decreased upon locust isolation (G*_isol_*) and increased upon crowding (S*_crowd_*). (F) DA-3S injection solicited locust aggregative behavior and enhanced the aggregation induced by DA. Four brain injections in saline solution were tested: Control (only saline); DA-3S (1 ng per brain); DA (10 ng); DA & DA-3S (1 ng DA and 10 ng DA-3S). The data are shown as mean ± SEM. ^*^, *P* < 0.05; ^*^^*^, *P* < 0.01; ^*^^*^^*^, *P* < 0.001.

We next attempted to identify the specific bioamine substrates catalyzed by SULT09225 in the behavioral changes. We tested the sulfation of three monoamine neurotransmitters (DA, 5-serotonin and tyramine) by recombinant expressed SULT09225 as the three bioamines have been validated to be highly important for locust neuronal and behavioral functions [5,8,9], as well as preferentially conjugated by catecholamine SULT [18,19]. The colorimetric assay revealed that SULT09225 only catalyzed the sulfation of DA but not the other two monoamines (Fig. 4A). Furthermore, the sulfate conjugation of DA catalyzed by SULT09225 occurred in a DA dosage-dependent manner (Fig. 4B). Thus, DA is an active substrate sulfated by SULT09225.

Meanwhile, we measured the levels of the three neurotransmitters and their sulfate conjugates in the locust brains. We biochemically synthesized the monoamine sulfates as reference standards and measured the brain contents of the free and conjugated monoamines using liquid chromatography-mass spectrometry (LC-MS) (see *Materials and Methods*). We examined four sulfate metabolites: dopamine-*O*-3-sulfate (DA-3S) (Fig. 4C), dopamine-*O*-4-sulfate (DA-4S), 5-serotonin-sulfate and tyramine-4-*O*-sulfate. We only detected DA-3S in the brain, which presented a concentration up to 10% of that of DA (Table S1).

We then determined whether *SULT09225* expression affects DA sulfation in locust brain using RNAi followed by LC-MS. *SULT09225* knockdown did not alter DA levels, but significantly reduced the content of DA-3S (*P* = 0.040) as well as the relative proportion of DA-3S to DA in the locust brain (*P* = 0.012; Fig. 4D). Therefore, SULT09225 catalyzes DA sulfation *in vitro* and *in vivo* of the locust brains.

We then examined how sulfation modification of DA affects behavioral aggregation of the locust. We first measured DA-3S production in response to population density changes. The brain content of DA-3S decreased 3.7-fold immediately upon locust isolation and increased 0.6-fold upon crowding, while the content of DA only marginally reduced upon isolation (*P* = 0.051) and remained unchanged upon crowding. The proportion of DA-3S relative to DA also exhibited a positive density response, implying an expedited sulfation of DA in aggregation (Fig. 4E).

We next investigated behavioral effects regulated by DA sulfation. Behavioral assays showed that DA-3S drove prompt behavioral transition toward aggregation in 1 hour after brain injection (*P* < 0.001). Furthermore, DA-3S enhanced the aggregation induced by DA (DA versus control: *P* = 0.006; DA-3S versus DA: *P* = 0.030; Fig. 4F). Thus, the DA sulfate efficiently promoted behavioral aggregation in the locusts.

### Sulfation in brain processes regulates behavioral plasticity in *C. elegans* and mouse

**Figure 5.**
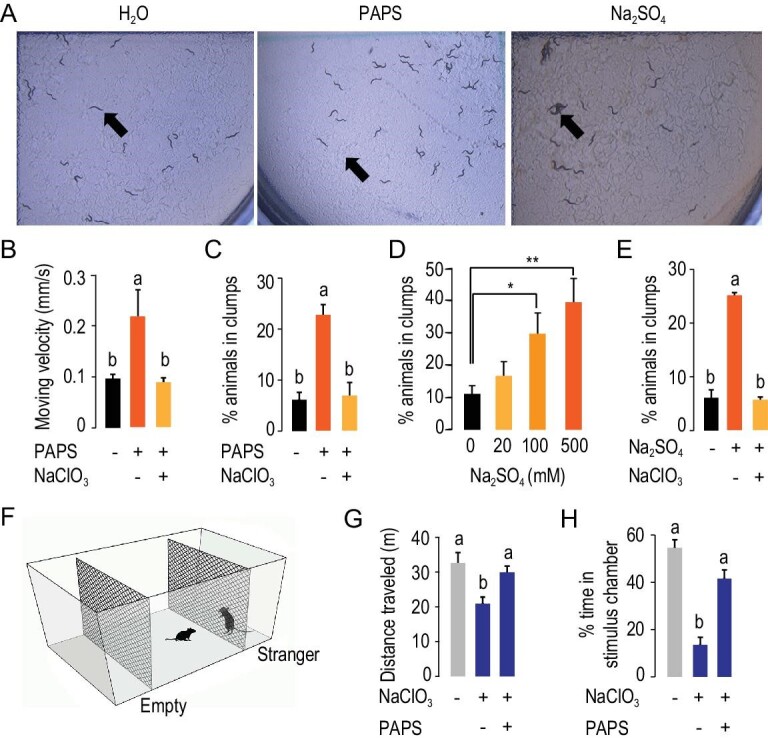
Pharmacological intervention of PAPS biosynthesis and sulfation modulates behavioral plasticity in *C. elegans* and mouse. (A) Aggregation behaviors measured in *C. elegans*. Two behavioral traits were measured: locomotion measured by the individual velocity of movement and aggregation behavior measured by the percentage of animals that clumped at 4 hours upon pharmacological treatment. A clump was defined as two or more animals that were in contact along at least half their length. Assay number of animals: n = 30 for locomotion; n ≥ 120 for aggregation. (B and C) Effects of PAPS supply on behavioral plasticity in *C. elegans*. Exposure to excess PAPS promoted animal aggregation and locomotion; NaClO_3_ culture after PAPS exposure rescued normal animal aggregation and locomotion. (D) Effect of sulfation reaction on behavioral plasticity in *C. elegans*. Sulfatase inhibitors, Na_2_SO_4_, promoted behavioral aggregation in a dosage-dependent manner in *C. elegans*. (E) Pharmacological intervention of sulfation reaction caused changes in aggregation behaviors in *C. elegans*. Na_2_SO_4_ feeding followed by NaClO_3_ culture rescued animal aggregation behavior. (F) Illustration of the three-chambered social testing apparatus used to measure mouse locomotion and sociability. Locomotion was measured by the total distance traveled in an open-field arena. Sociability was measured by a three-chamber social approach and quantitated by exploration time in one side attraction area neighboring the stranger chamber containing a stimulus male mouse versus the total time in the attraction area and the opposite area neighboring the empty chamber. (G and H) Pharmacological intervention of brain PAPS supply affected mouse locomotion (G) and sociability (H). NaClO_3_ injection reduced mouse locomotion and sociability; PAPS injection after NaClO_3_ injection rescued normal mouse locomotion and sociability. Intracerebroventricular infusion of NaClO_3_ and PAPS was performed. n = 10 per group. The data were shown as mean ± SEM. Student *t* test: ^*^, *P* < 0.05; ^*^^*^, *P* < 0.01; ^*^^*^^*^, *P* < 0.001.

As the sulfation system is highly conserved in animals [19], we examined whether sulfation is also involved in regulating behavioral plasticity in model systems such as *C. elegans* and mouse. We first investigated the role of PAPS biosynthesis in the regulation of two plastic behaviors in the *C. elegans* N2 strain, that is locomotion, as measured by animal moving velocity in culture media, and social aggregation, as measured by the percentage of animals in a population that clumps at 4 hours after treatment (Fig. 5A). PAPS administration significantly increased individual moving velocity (*t* test: *P* = 0.002, Fig. 5A and *B*). And the effect from an overdose of PAPS was significantly repressed by chlorate administration, which inhibits PAPS synthesis (*P* = 0.032, Fig. 5B). *C. elegans* individuals of N2 strain typically exhibited solitarious behaviors as described [6]. PAPS administration increased the percentage of animals in clumps 2.8-fold (*P* = 0.010, Fig. 5C). Additional chlorate administration significantly reduced the percentage of animals in clumps from 23% to 7% (*P* = 0.006, Fig. 5C). Thus, the biosynthesis and availability of PAPS efficiently regulate locomotion and aggregative behaviors in *C. elegans*.

We next investigated whether the processes of sulfation/desulfation are involved in behavioral regulation in *C. elegans*. The sulfatase inhibitor, Na_2_SO_4_, was used to inhibit the desulfation reaction, thus likely promoting sulfation processes [20]. Na_2_SO_4_ solicited animal clumping, and exhibited a dose effect in the range of 0–500 mM (*P* < 0.05, Fig. 5A and D). Then we tested whether the behavioral effects from desulfation could be repressed by limiting PAPS supply through chlorate administration. The chlorate feeding can greatly reduce effects on animal aggregation (*P* < 0.001, Fig. 5E). Taken together, the catalytic pathway from PAPS biosynthesis to sulfation plays a critical role in regulation of behavioral plasticity in *C. elegans*.

Lastly, we investigated the effects of brain PAPS availability on mouse behaviors in terms of locomotion and sociability (Fig. 5F, also see *Materials and Methods*). NaClO_3_ injection in mouse brain through intracerebroventricular infusion caused a potent inhibitory effect on expression of two *PAPSS* genes, *PAPSS1* and *PAPSS2*, in three brain tissues, the hippocampus, hypothalamus and olfactory bulb (Fig. S7). NaClO_3_ significantly reduced mouse locomotor activity in an open field chamber (*P* = 0.011, n = 10). Additional PAPS administration partially rescued the effect on locomotor activity (*P* = 0.036, n = 10) (Fig. 5G). Sociability was quantitated by measuring the exploration time in one of two side chambers containing an unfamiliar conspecific (stimulus) in a wire cage in comparison with the total time in the stimulus and the opposite empty side chamber [2] (Fig. 5F). Thus, sociability represented the preference of mice for initiating social interactions with novel conspecifics [2]. NaClO_3_ administration significantly repressed the social preference for conspecifics (*P* < 0.001, n = 10). Additional PAPS dose rescued mouse sociability (*P* < 0.001, n = 10) (Fig. 5H). Thus, PAPS availability in brain modulated mouse behavioral plasticity.

## DISCUSSION

Here we have reported that sulfation modification in brain is a potent molecular process modulating animal behavioral plasticity and the neurotransmitter DA is predominantly sulfated for the behavioral regulation. Previous studies have shown that sulfation processes are involved in several biological functions, such as detoxification, development and physiology [19,20,30,31]. Large biomolecules such as proteins and glycosaminoglycans are sulfated mainly for developmental and physiological regulation [20,21], including neural plasticity and progressive neurodegeneration [25]. Steroid sulfation and desulfation pathways are highly relevant in the regulation of steroid hormone action and are involved in a multitude of endocrine-related pathologies [32]. However, brain-related sulfation and its relevance to behavioral regulation have been largely ignored. In this study, we revealed that the sulfation modification regulates the behavioral aggregation associated with changes of population density in the locust. Notably, chemical inhibition of the sulfation process can reverse the behavioral phase of the locust within 1 hour. As a contrast, addition of sulfate donor PAPS enhances the aggregative behavior. Note that several studies provided evidence that PAPS can translocate across some cells, possibly through GAP junctions, or can affect sulfation from the extracellular spaces [33,34]. Extracellular PAPS could also be provided to Golgi-resident SULTs through unknown mechanisms. Alternatively, PAPS could work in the extracellular space as a sulfate donor of novel sulfation process, or through the action of an unknown PAPS transporter [33,34].

Sulfation represents phase II metabolism and serves as a highly efficient way for molecular modification [25]. The major class of sulfated molecules involved in rapid behavioral response could be small compounds such as catecholamines. We exclusively examined social or social-like behaviors which are particularly plastic in animals. However, this type of modification is conserved as a regulatory mechanism across animal species from invertebrates to vertebrates, even though the specific modified molecules regulating these behaviors may have diverged in different types of behaviors and among species. These findings support our hypothesis that sulfation may act as a common regulator of behavioral plasticity across animal species, and renew our understanding of the elaborate modulation of animal behavioral response to environmental stimuli.

Sulfation in the brain regulates behavioral plasticity, possibly through sulfation-mediated molecular rewiring of neuronal systems. We showed that some catecholamines could be preferentially sulfate conjugated in locust phase transition. The discovery was verified by the fact that injection of the SULT inhibitor PAP drove a dramatic reversion of behavioral aggregation. We further identified a bioamine SULT, SULT09225, that catalyzes DA sulfation *in vitro* and in the brain. SULT09225 belongs to the subfamily phenol SULT and is an ortholog of human catecholamine SULT. Such SULT contains a unique glutamate ‘E’ in the substrate-binding site, specifically binding catecholamines such as epinephrine, norepinephrine and DA [32]. Our study verified the substrate DA catalyzed by SULT09225, but did not exclude the involvement of other targets except serotonin and tyramine. *SULT09225* expression knockdown repressed the behavioral aggregation that involves changes of locomotion and attraction of the locust. Interestingly, a mutation of *SULT* (*SULT4A1*) also reduces zebrafish daytime activity and conspecific attraction [24], and inhibition of a steroid sulfatase enhances mouse memory and aggressive behaviors [31]. These results indicate that catecholamine SULT and other phenol SULTs are involved in behavioral regulation in different animal models. Nevertheless, Alcian Blue staining revealed different levels of sulfated molecules, including sulfated polysaccharide, in the brain of G and S phase (Figs 1 and 2). Our results thus did not exclude the possible involvement of sulfated polysaccharide in the behavioral regulation, for example, in an indirect way by interacting with neurotransmitter receptors [35].

This study provides the first instance that the key neurotransmitter DA in brain is chemically modified through sulfation to mediate behavioral aggregation. Most portions of DA in biles and other organs are present as its sulfate conjugates [36]. However, because of the blood-brain barrier (BBB), only a small percentage of the trace DA in the brain is sulfated, although DA sulfate can permeate the BBB in small amounts [37]. Among the isomers of DA, we only detected DA-3S but not DA-4S in the locust brain. DA-3S is also present and predominant over DA-4S in the human brain [38,39]. In fact, the isoenzyme SULT1A3 is regioselective and strongly favors formation of DA-3S over DA-4S in the human brain [39]. It is widely believed that DA is sulfated for degradation in these tissues. However, our study revealed that sulfate conjugation of DA in locust brain is tightly regulated by population density and promotes behavioral aggregation in the locusts. Furthermore, DA sulfate causes more rapid and prominent behavioral changes than free DA. Although the roles of DA in the regulation of locust behavior have been recognized [8,10,11], the sulfated DA was not separately measured relative to free DA in those studies. Sulfated DA levels mediate aggregative behaviors in locusts that involve both locomotion and attraction or sociability-like predisposition. However, it seems that the motivation to be aggregative or social is affected to a larger extent than locomotion by DA sulfation. Similar effects observed with the general sulfation processes corroborate this result (Figs 2 and S3). The results here highlight the importance of DA sulfation metabolism for animal behavioral regulation.

Several mechanisms may explain how DA sulfation alters DA signaling and causes the observed behavioral changes. First, DA sulfate conjugation could lead to an altered interaction with a subclass of DA receptors. Some studies suggested that sulfate conjugation causes inactivation of DA [38]. However, there are two types of DA receptors, that is activating (D1-like) and repressive (D2-like) receptors. Also in the locust, DA receptors (Dop1 and Dop2) function with contrasting signaling effects on the aggregation behaviors [10,11]. The DA metabolites (e.g. DA-3S, DA-4S) could be biologically ineffective at central D2 receptors [40]. Thus, DA sulfate may attenuate the repressive effects of certain DA receptors, while DA enhances their signaling effects. Secondly, sulfation is a bioactivation step for some molecules and in certain signaling pathways [41]. DA sulfation affects NMDA receptor signaling by DA-receptor interaction. DA can also induce upregulation of SULT1A1 and SULT1A3 that catalyze DA sulfation [42]. Therefore, the previous behavioral effects from DA may involve the sulfation metabolism of itself. Thirdly, the sulfate metabolite of DA may serve as a reservoir of DA *in vivo*, contributing indirectly to the biological effects in brain. DA sulfate conjugate has been suggested to serve as a transport form of DA into cells, where free DA could be regenerated. Lastly, but not exclusively, the plastic response of the neuronal system could be mediated or even enhanced through homeostatic tuning of sulfated versus unsulfated DA [25], or through a relocation of its metabolic pathway (e.g. to a norepinephrine precursor). In all, the specific mechanisms for dopamine sulfate-altered dopamine signaling need further investigation.

Our findings shed light on the molecular basis of some important neuronal and behavioral diseases. Natural mutations or deficiency in sulfation pathways have been implicated in the progression of neurodegenerative diseases and behavioral disorders associated with motor, learning and memory [22,23,31,32]. The copy number of SULT1A3/4 is associated with Parkinson's and Alzheimer's disease [43]. A genetic link has been found between mutations in the SULT1A locus and autism [23]. Thus, a major goal of future study is searching for more neurotransmitter therapy targets that could be sulfation modified in human behavioral and neuronal diseases.

## MATERIALS AND METHODS

### Insects

The gregarious (G) and solitarious (S) locusts were reared in the laboratory at the Institute of Zoology, Beijing, using standard methods [8,26]. Briefly, G locusts were cultured in a large cage (40 × 40 × 40 cm) from new hatchlings at a density of approximately 400 individuals per cage. G locusts had been reared for more than five generations and served as the source for derived S strains. S locusts were cultured individually in white metal chambers (10 × 10 × 25 cm) supplied with circulated fresh air. S nymphs had been reared for three to four generations before experiments. All strains were fed on fresh wheat seedlings and bran and maintained under a 14 : 10 hour (L : D) photoperiod at 30 ± 2°C. The behaviors of locusts in G and S phase are reversible. However, it is much more difficult for locusts to complete a full behavioral transition from solitaria to gregaria than vice versa [4,8,26]. Because 4^th^-instar nymphs exhibited most prominent phase-specific behavioral traits and expression of genes and metabolites [3,4,8], individuals at this stage were sampled and tested in this study.

### Immunoblotting assay

Affinity-purified polyclonal antibodies against PAPSS were developed by BGI (Beijing, China) and used at 1 : 200 dilution. Tubulin (1 : 10 000 dilution, laboratory prepared) or GAPDH (1 : 5000 dilution, CoWin, Beijing) was used as the internal control for determining total protein amounts. Samples of 10 brains were collected for each of three biological replicates.

### Alcian blue staining

Alcian Blue is a cationic histochemical stain, used widely for *in situ* detection of sulfated molecules [44]. Whole brain was fixed *in situ* with 4% paraformaldehyde (Sigma-Aldrich, USA) overnight. Then the tissue was embedded in 2% low melting point agarose in an embedding block. After the agarose hardened, embedded brain was trimmed and dissected into 40 μm using Leica VT1200S vibratome (Leica, Germany). The brain slices were stained with 0.01% Alcian blue 8GX (Sigma-Aldrich, USA) in 3% acetic acid, pH = 1.0, and shaken for 3 hours. The stained brain was then rinsed with H_2_O for 2 hours and imaged under a Leica DM2500 microscope (Leica, Germany). The dye 4´,6-diamidino-2-phenylindole was applied to counter-stain the nuclei of the overlying cell body layer (data not shown).

### Gene cloning, sequence alignment and phylogenetic analysis

To obtain locust *PAPSS* and *ST* genes, we searched the gene annotations of the locust genome database as well as blasted their human ortholog against the locust genome and transcriptome sequence databases (www.locustmine.org). Full-length cDNA sequence of *PAPSS* was obtained by 5^′^ RACE and 3^′^ RACE using the SMART™ RACE cDNA Amplification Kit (Clontech, USA) according to the manufacturer's instructions. The cDNA sequences of locust genes have been deposited in GenBank under the following accession numbers: KT000398 (*PAPSS*), KT000399–KT000414 (the 16 locust *ST*s).

Alignments of amino acid sequences were constructed using MEGA6 [45]. Construction of a phylogenetic tree using amino acid sequences were performed by the Neighbor-Joining methods. Gaps were excluded based on pairwise distance estimation. Reliability of the trees was tested by a bootstrap procedure with 1000 replications. GenBank accession numbers for the sequences used in analysis were as follows: H. sapiens_PAPSS1, NP_005434.4; H. sapiens_PAPSS2, NP_004661.2; M. musculus_PAPSS1, NP_035 993.1; M. musculus_PAPSS2, NP_035994.2; D. melanogaster_PAPSS, AAN11638.1; C. elegans_PPS-1, CAA93098.1; L. migratory_PAPSS, KT000398; the 16 *L. migratoria* STs, KT000399 - KT000414; Human SULT1A1, AAI10888.1; SULT1A2, NP_803564.1; SULT1A3, NP_808220.1; SULT1A4, NP_001017390.1; SULT1B1, NP_055280.2; SULT1C1, AAF72804.1; SULT1E1, NP_005411.1; SULT2A1, ACB21041.1; SULT2B1, NG_029063. The sequence motifs characteristic of STs were identified based on human STs [18]. All primers used and the name and GenBank accession number of all the genes are listed in Table S3.

### Quantitation of gene expression by real-time quantitative PCR

Brain tissues of locusts or mice were dissected and immediately stored in liquid nitrogen until RNA isolation. Each sample contained 10 locust brains or one mouse brain. RNA was purified using TRIzol (ThermoFisher, USA) according to the manufacturer's instructions. The methods for reverse transcription and qPCR followed Chen *et al.* (2015). The PCR program was 95ºC for 2 minutes, followed by 45 cycles of 95ºC for 20 seconds, 58ºC for 20 seconds and 68ºC for 20 seconds using the SYBR Green kit and performed on a LightCycler 480 instrument (Roche). The melting curve was analyzed to confirm the amplification specificity of the target gene. All PCR amplifications were sequenced to verify the specificity of primers. Ribosomal protein 49 (*Rp49*) and *GAPDH* were used as the internal control in the locust [11,46] and mouse, respectively. All reactions were performed in triplicate. Relative expression was quantified using the 2^–ΔΔCt^ method. Primers are listed in Table S3.

### RNA interference (RNAi)

Sequences of dsRNAs were tested for their specificity of interference by blasting their sequences against the locust genome database. dsRNA of target genes and control gene, that is green fluorescent protein (*GFP*), were prepared using the T7 RiboMAX Express RNAi system (Promega, USA) following the manufacturer's instructions. Before dsRNA injection, the 2-day-old 4th-instar nymphs were first mounted on a Kopf stereotaxic frame specially adapted for locust surgery. The nymphs were injected with 69 nL of dsRNAs (500 ng/μL) into brain. The injected locusts were then marked and returned back to their rearing cage or box and supplied with food. The nymphs were reared for 24 hours or 48 hours before tissue collection or behavioral assay. Five biological replicates of eight nymphs (four males and four females) were examined for each treatment in expression assay. Primers for dsRNA synthesis are listed in Table S3.

### Behavioral assay of the locust in arena

Locust behaviors were measured in a rectangular Perspex arena (40 × 30 × 10 cm) according to a method developed by Roessingh *et al.* [47] and improved in several studies [3,8,9,26]. After a locust was released into the arena, its behaviors were monitored for 300 seconds and analyzed using an EthoVision video tracking system (Noldus Information Technology, Netherlands). Behavioral phase states of locusts were defined using an optimized binary logistic regression model: *P*_greg_ = −2.11 + 0.005 × AI + 0.012 × TDM + 0.015 ×TDMV (AI, attraction index (s); TDM, total distance moved (m); TDMV, total duration of movement (s)). AI stands for the propensity of tested animals attracted by the stimulus group and is calculated as: AI = Total duration in stimulus area (s) − Total duration in the opposite of stimulus area (s). This model correctly classifies 87.2% of gregarious populations and 87.0% of solitarious populations [26]. Note that the sample number may vary with experiments even though we started rearing a batch of approximately the same number of newly hatched nymphs for a behavioral assay. We only selected 2-day old fourth-instar nymphs for the assay. To minimize the effects of daily rhythm, we performed the behavioral assay in the afternoon and finished comparative experiments in two consecutive days. During the experiments, a few nymphs were exceptionally tardy, and were not moving in the arena and thus were discarded for the final analysis.

### Pharmacological intervention in locusts

Chlorate is known to be an effective inhibitor of ATP-sulfurylase (i.e. PAPSS), causing reversible inhibition of PAPSS but not affecting cell growth or protein synthesis [27,28]. The inhibitory potency and specificity of chlorate to sulfation was validated in the locust (Fig. 2E, 2F 3D and Fig. S2). Sodium chlorate (NaClO_3_) was purchased from Sigma-Aldrich and dissolved into water. A total of 3 μL sodium chlorate (0, 20 and 200 mM) was injected into the second ventral segment of G nymphs’ abdomen. Then, the injected locusts were marked and put back into gregarious-rearing cages. Behaviors were assayed at 1 hour after the injection of 3 μL 200 mM NaClO_3_ or 3 μL ddH_2_O (i.e. control).

PAPS has been considered as a universal and obligate sulfate donor in all eukaryotes. PAPS was purchased from Sigma-Aldrich (Cat. # A1651) and dissolved into ddH_2_O. A total of 4 μL PAPS (10 nM) or 4 μL ddH_2_O (i.e. control) was injected into the second ventral segment of S nymph abdomen. Then, the injected locusts were put back into solitarious-rearing boxes. At 1 hour and 4 hours later, the behavior of nymphs was examined.

PAP is a potent competitive inhibitor of SULTs, and most effective for inhibiting the transformation of dopamine and phenol substrates [18,29]. PAP was purchased from Sigma-Aldrich and diluted in ddH_2_O into 10 nM for use. The method for PAP injection was the same as that used for NaClO_3_ in behavioral assay. The behavior of nymphs was examined at 1 hour after the injection.

### Behavioral assay of locust in Y-tube

Y-tube olfactometer was used to analyze the olfactory behavior of individual locusts as described previously [5]. Volatile odorants represented volatiles from body and feces from 30 G locusts in the absence of any visual cues. The air flow was set at 300 mL/min. An individual locust was recorded as ‘first choice’ for volatile or fresh air whenever the locust moved more than 5 cm into either arm in 4 minutes. The percentage of locusts that chose either the volatile arm or the control arm was used to quantify the choice behavior.

### Recombinant expression and purification of SULT09225

The coding sequence of SULT09225 was amplified from locust cDNA using the gene-specific primers (see primer sequences in Table S3). The PCR product was ligated into EcoRI and XholI sites in the PET28a expression vector using T4 DNA Ligase. The protein was then recombinantly expressed in *Escherichia coli* DE3. The induced bacteria was broken by the ultrasonic wave and the supernatant was passed over a HisPur Ni-NTA column at 4°C. The eluate was collected separately and analyzed using SDS-gel electrophoresis. The purified protein was ultra-filtered before sulfation activity assays.

### SULT enzyme activity assay

The enzyme activity of SULT09225 was measured using a colorimetric assay that was originally developed for SULT1A1 activity assay with human samples [48]. Substrate receives a sulfonate from PAPS under catalysis of SULT generating substrate sulfate and PAP. At the same time, PAP promotes p-nitrophenyl sulfate in the solution to transform into p-nitrophenol, which can be captured and quantified by 405 nm UV. The standard reaction mixture consisted of 50 mM potassium phosphate buffer (PBS, pH 6.5), 5 mM magnesium chloride, 20 μM PAPS (Cat. #ES019, purity >90%, R&D Systems, USA), 5 μM *p*-nitrophenyl sulfate, monoamine substrates, and the recombination SULT09225 with a final volume of 1 mL in a 96-well microtiter plate. Gradient concentrations of DA (Sigma), serotonin (Sigma) and tyramine (Sigma) were used to test the substrate specificity of SULT09225. The reactions were allowed to proceed for 8 hours at 37°C. Then the relative absorbance at 405 nm UV was analyzed on the Muti-Mode Detection Platform (SpectraMax^®^ Paradigm^®^) every hour. Three replicate assays of three replicate samples in each treatment were performed.

### Liquid chromatography-mass spectrometry (LC-MS)

The methods for sample preparation were improved from reported protocol [49]. Specifically, brains were immediately dissected and stored in liquid nitrogen before assay. Twenty brains from 10 males and 10 females were collected for each of at least five biological replicates. Each sample was homogenized for 1 minute with 400 μL of ice-cold 0.1 M perchloric acid containing 10^–7^ M ascorbic acid. The homogenate was centrifuged at 5200 g for 4 minutes at 4ºC. The supernatants were filtered through 0.22 μm filters (Millipore, USA). Then an equal volume of methanol was added to the filtered sample before LC-MS assay.

The assay on PAPS content in brain was performed on Agilent 1200 (Santa Clara, USA) and API 4000™ LC-MS/MS System (ABI SCIEX, USA) using Selected Reaction Monitoring (SRM) with positive ion mode. HPLC fractionation was conducted in the column Chromolith FastGradient RP-18e 50–2 mm (Merck, USA). The mobile phase system was A: aqueous 0.1% formic acid (Sigma-Aldrich, USA) and B: acetonitrile (J&K, China) plus 0.1% formic acid. The linear gradient was B 30% 0–0.4 minutes, 100% 0.45–2.5 minutes, 30% 2.8 minutes, followed by a 3.8-minute equilibrium. The flow rate was adjusted to 0.8 mL/min, and the temperature was set at 35°C. Tolbutamide was used as the internal standard. The SRM pair used for PAPS and tolbutamide was *m/z* 508/207 and *m/z* 271.3/171.8, respectively. Peak area ratios (PAPS/IS) were plotted against the concentration (ppb, ng/mL) of the analytes injected. The injection volume is 1 μL. The standard curve was prepared with PAPS (Cat. #ES019, R&D Systems).

Before the measurement of free monoamines and their sulfates, the sulfated monoamines, dopamine-*O*-3-sulfate, dopamine-*O*-4-sulfate, tyramine-4-*O*-sulfate and 5-serotonin-sulfate, were first chemically synthesized and used as reference standards [50]. The detailed methods for the chemical synthesis of these sulfates were available at request. The free amines were purchased from Sigma-Aldrich (USA). The assay was performed on an Agilent 1260/6460 HPLC-triple quadrupole mass spectrometer (Santa Clara, USA) using SRM. Calibration standard was prepared in Ringer's solution and was run as an external standard [38,50]. The HILIC separation was performed using a ZIC-CHILIC column (2.1 × 150 mm, 3 μm-) together with a ZIC-CHILIC guard column (2.1 × 20 mm) (Merck SeQuant, Germany). The mobile phase system was A: aqueous 0.12% (v/v) formic acid (Sigma-Aldrich, USA), containing 2.5% (v/v) methanol (Thermo Fisher, USA) and 5 mM HCOONH_4_ (Agilent, USA), and B: acetonitrile (Thermo Fisher, USA). The linear gradient was B 90% 0–2 minutes, 90%–80% 2–32 minutes, 80%–30% 32–34 minutes, 30% 34–38 minutes, 30%–90% 38–40 minutes, 90% 40–56 minutes. The flow rate was adjusted to 0.27 mL/min, and the temperature was set at 35°C. The injection volume was 5 μL.

For the LC-MS/MS analysis, ESI was performed in positive ionization mode for tyramine, 5-serotonin, DA and in negative ionization mode for the four sulfated bioamines. The flow rate of drying gas was 12 mL/min, the atomizing gas pressure was 35 psi and the temperature of solvent removal was 350°C. Nitrogen was used as collision gas. The capillary voltage was 4.0 and 3.5 kV for positive mode and negative mode, respectively. Under the LC-MS analysis system, the seven examined compounds above were eluted at specific retention time (Table S1). The fragmentor voltage and collision energy for different SRM pairs were optimized for each compound. Mass spectrometric detection in an ESI positive mode was conducted using SRM with the different reaction condition for each compound (Table S1). Data in the positive and negative ion mode were acquired in separate runs.

### Behavioral assay of *C. elegans*

We characterized the plastic behaviors of *C. elegans* using two traits following published methodologies [6]: aggregation behavior by measuring the percentage of animals in a population that clumps at 4 hours after treatment, and locomotion by measuring moving velocity of individuals in culture media. *C. elegans* worms used in the experiments belong to the canonical wild-type strain N2 that exhibits solitarious behaviors [6]. NGM culture plates containing 2.1% agar were evenly seeded with 200 μL of OP50 *E. coli* in NGM medium.

To introduce the pharmacological inhibitor, Na_2_SO_4_ or NaClO_3_, one of the chemicals was added to an *E. coli* solution to obtain a final preset concentration. Sulfate ions (SO_4_^2–^) are competitive inhibitors of arylsulfatases [20,31] and thus used for desulfation inhibition. The effects of Na_2_SO_4_ (Beijing Chemical Co., China) on aggregation behavior were tested at a range of concentrations (0–500 mM). The concentration of 500 mM Na_2_SO_4_ was adopted for the subsequent experiments. Two days later, more than 120 well-fed adult worms from uncrowded plates were picked onto this lawn and cultured at 20ºC for 4 hours. As PAPS is easy to degrade at non-freezing temperature, we introduced PAPS into animals by submerging worms into *E. coli* solution containing 20 nM PAPS for 20 minutes at 20ºC. Then the animals were transferred to a new plate for behavioral assay. Rescue experiments were performed by transferring those animals pretreated in PAPS or Na_2_SO_4_ to the new plate containing 200 mM NaClO_3_ or H_2_O (control) and culturing at 20ºC for 4 hours before behavioral assay.

Clumping behavior was measured by calculating the fraction of animals that were in contact with two or more other animals along at least 50% of their body length. At least three replicates of 120 worms were used. To quantify locomotion, a well-fed adult worm was transferred to new plate with food and left undisturbed for 5 minutes. Movement was then recorded for 100 seconds using a digital camera that was mounted onto a dissecting microscope (M205C, Leica, Germany). Video recordings were carried out at room temperature. Videos of worms were processed using the EthoVision video tracking system (Noldus Information Technology, Netherlands). Thirty animals were examined for each treatment.

### Stereotaxic surgery for intracerebroventricular infusion in mice

Mice (line C57BL/6) were reared in isolation from postnatal day (P) 60 under a 12/12 hour light/dark cycle in a temperature and humidity-controlled environment with free access to food and water. Each P70 male mouse was subjected to cannula implantation under aseptic conditions. Animals were anesthetized by intraperitoneal injection of a mixture of ketamine and xylazine. For intracerebroventricular infusion, animals were implanted unilaterally with a 26-gauge guide cannula (RWD, China) targeted to the lateral ventricle (from the bregma: −0.58 mm anteroposterior, −1.0 mm mediolateral, −1.5 mm dorsoventral) using a standard stereotaxic technique. The implanted cannula (RWD) was cemented to screws on the skull. A 33-gauge dummy cannula (RWD) was inserted into the guide cannula during recovery. Animals were left in the home cage for 8 days for recovery. All animal experiments were conducted in accordance with the guidelines of the Animal Care and Use Committee of the Institute of Zoology, Chinese Academy of Sciences.

### Behavioral assay of mouse

To start the test, each animal was individually placed into the open field and allowed to explore the arena for 30 minutes for environmental acclimation. After 30 minutes, the animals were quickly removed from the apparatus and injected with 4 μL 1 M NaClO_3_ or 4 μL sterile 0.9% NaCl saline solution (control) within 2 minutes. The inhibitory potency of chlorate to sulfation was validated in mouse (Fig. S7). Following the administration, the animals were immediately placed back into the same open-field arena and allowed to explore freely for 60 minutes. Then the behaviors of each animal were recorded for 10 minutes. To rescue sulfation, the animals were injected with 4 μL 10 nM PAPS or 4 μL 0.9% NaCl saline solution (control) at 60 minutes after the chlorate infusion. The animals were immediately placed back into the same open field arena and allowed to explore freely for 60 minutes. Then the behaviors of animal were monitored for 10 minutes.

We measured locomotor activity of mouse in an open-field arena (50 × 30 × 20 cm) made of white non-translucent Plexiglas. Locomotor activity was quantitated as the total distance traveled in 10 minutes. The methods used for mouse sociability followed the established three-chamber social procedure with some improvements [2]. The middle chamber has a width four times that of either side chamber. After the habituation period in the three-chambered testing apparatus, an unfamiliar male mouse, which had no prior contact with the subject mice, was placed in a wire cage in one of the side chambers to act as stimulus for social interaction. The subject mouse was allowed to explore the social chambers for 10 minutes. The mouse trail was recorded with automated tracking software (EthoVision, Noldus, Netherlands). In trail analysis, the middle chamber is evenly divided into four areas, that is one attraction area neighboring the stranger chamber, two non-choice areas in the middle and one repulsion area neighboring the empty chamber. Sociability was quantitated by exploration time in the attraction area versus the total time in the attraction area and the opposite repulsion area. The chambers were cleaned, and the stimulus and control chamber were alternated between trials. Experiments were conducted during the animal's dark phase. Each animal was only exposed to one unique behavioral test. Ten mice were used in each treatment.

### Statistics

The frequency data of behavioral features *P*_greg_ and AI were analyzed with the Mann–Whitney *U* test. The counts of locust choice on the volatile or air arms were analyzed with Fisher's exact test to compare within treatment between air-volatile choices and a neutral 50% decision, or between treatments (NaClO_3_ vs. ddH_2_O). Independent-sample Student *t* tests were performed to compare difference in gene expression and other behavioral traits between treatments. Data were presented as mean ± SEM.

### DATA AVAILABILITY

All data needed to evaluate the conclusions in the paper are present in the paper and/or the Supplementary data. Additional data related to this paper may be requested from the authors.

## Supplementary Material

nwab163_Supplemental_File
