# Supplementary material for: Sulfation modification of dopamine in brain regulates aggregative behavior of animals
Source: Natl Sci Rev. 2021 Sep 2;9(4):nwab163. doi: 10.1093/nsr/nwab163 (PMC9072122; doi:10.1093/nsr/nwab163)
Supplement: nwab163_Supplemental_File [file nwab163_supplemental_file.docx]

**Sulfation modification of dopamine in brain controls aggregative behavior of animals**

Bing Chen, et al.

**Supplementary materials**

Fig. S1. Phylogenetic and expression analysis of *PAPSS* in the locust

Fig. S2. Knockdown of *PAPSS* expression results in a behavioral transition from gregarious to solitarious phases at 48 h after dsRNA injection in the locust

Fig. S3. Effects of PAPS on the behavioral traits of locust nymphs at 1 h and 4 h upon PAPS injection

Fig. S4. Expression of cytosolic *SULT* genes in the time course of locust isolation and crowding

Fig. S5. Multiple alignment of the locust sulfotransferase *SULT_09225* with human cytosolic SULTs

Fig. S6. Recombinant expression of SULT09225 and bioamine sulfation catalyzed by SULT09225

Fig. S7. NaClO3 infusion inhibits PAPSS expression in mouse brain

Table S1. Brain contents of the nine compounds and their parameters used for LC-MS/MS

Table S2. Expression of *SULT* genes in the locust brains

Table S3. Sequences of all primers used in the experiments


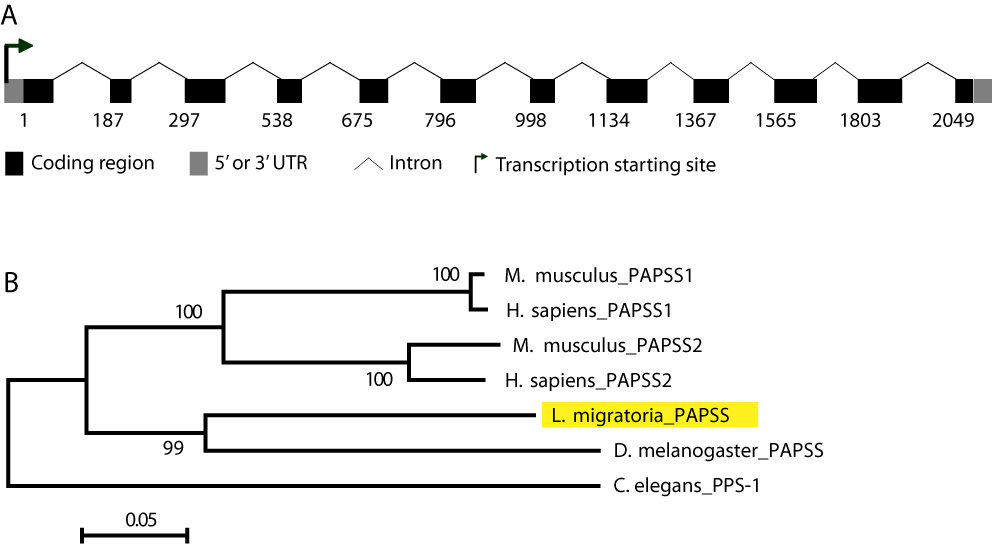


# **Fig. S1.** P**hylogenetic and expression analysis of PAPSS in the locust. (A)** Gene structure of the locust *PAPSS*. There is one *PAPSS* gene of 3,003 bp in the locust genome. Numbers represent the starting site of the 12 coding exons that putatively encode 715 amino acids of locust PAPSS. The catalytic domain of ATP sulfurylase is located in the carboxyl-terminal section, whereas the APS kinase domain is in the amino-terminal region of this bifunctional protein. **(B)** Phylogenetic analysis of PAPSS protein sequences. The numbers at the nodes represent bootstrap support values obtained by the neighbor-joining method. The scale bar represents 0.05 substitutions per site. The locust PAPSS is shaded in yellow. The GenBank accession numbers for these sequences are provided in *SI Materials and Methods*. Abbreviation for species name: *M. musculus*, *Mus musculus*; *H. sapiens*, *Homo sapiens*; *D. melanogaster*, *Drosophila melanogaster*; *C. elegans*, *Caenorhabitis elegans*.


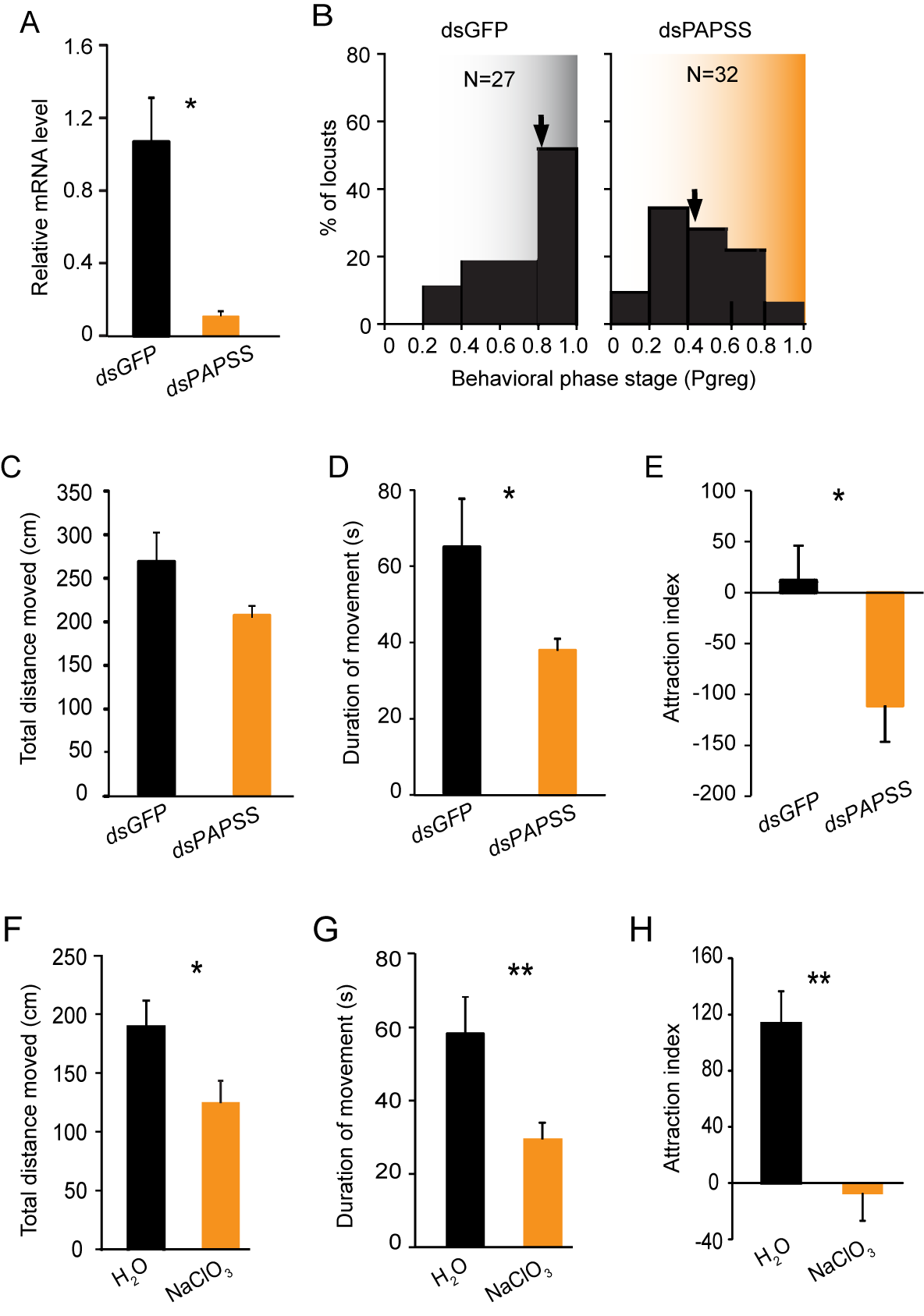


# **Fig. S2. Knockdown of *PAPSS* expression results in a behavioral transition from gregarious to solitarious phases at 48 h after dsRNA injection in the locust. (A)** RNAi reduced mRNA level of *PAPSS* in the nymphal brain at 48 h after dsRNA injection (*P* = 0.018). The mRNA level was quantified by qPCR and normalized against that of the internal control *RP49*. Five biological replicates of eight nymphs were examined for each treatment. **(B)** *PAPSS* interference resulted into a behavioral transition from gregaria to solitaria at 48 h after dsRNA injection (Mann-Whitney *U* test, *P* < 0.001). Arrows indicated median *P*_greg_ values. N denoted the number of nymphs measured for behavior. (**C-E**) Changes in behavioral traits caused by *PAPSS* knockdown at 48 h after ds*PAPSS* injection. The behavioral traits included the total distance moved in arena **(C)**, the duration of time of movement **(D)**, and the attraction index **(E)**. (**F, G**) NaClO_3_ injection caused a reduced total distance moved **(F)** and duration of time of movement in arena (***G***). (**H**) NaClO_3_ injection resulted into a conversion from an olfactory attraction to repulsion. The data are shown as mean ± SEM. Asterisks indicate the significance level: *, *P* < 0.05; **, *P* < 0.01.


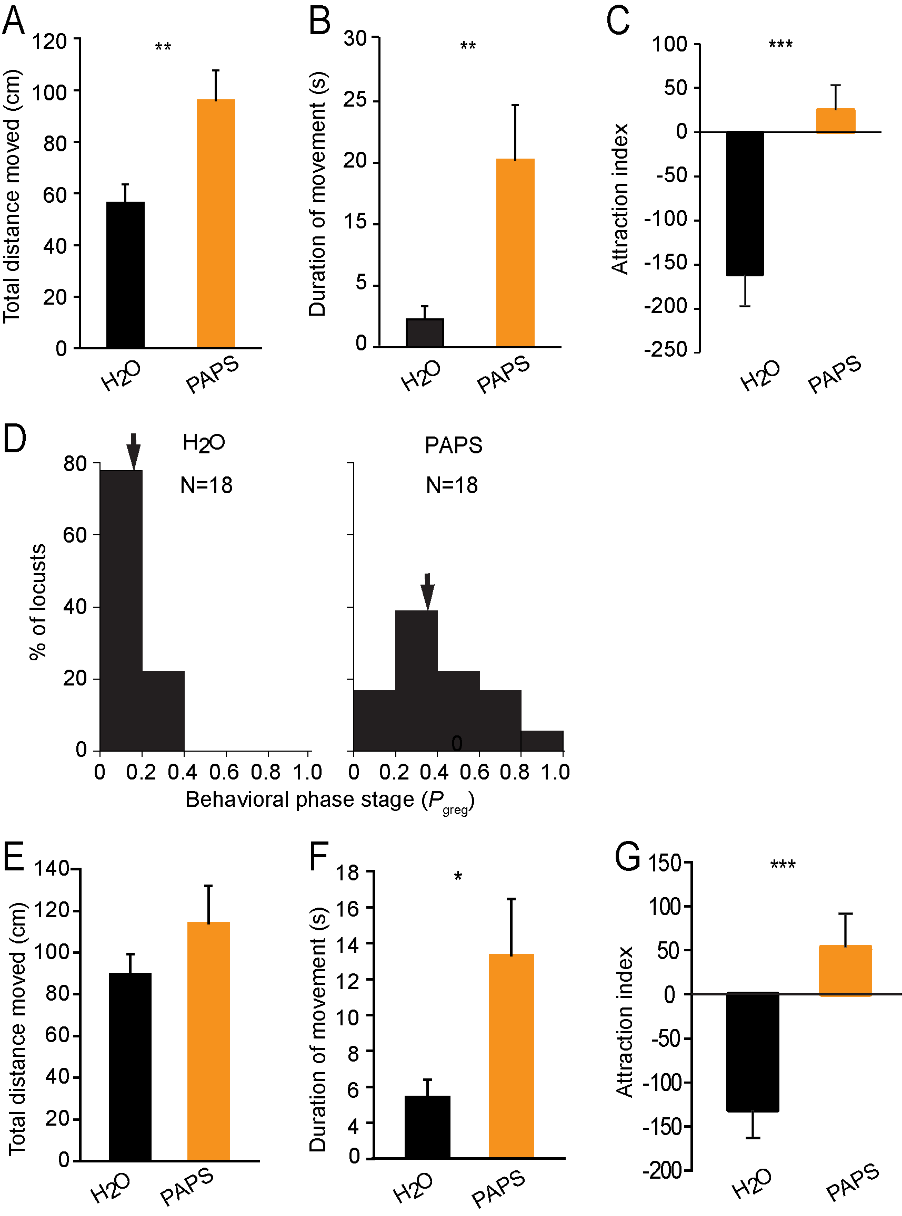


# **Fig. S3.** **Effects of PAPS on the behavioral traits of locust nymphs at 1 h and 4 h upon PAPS injection.** (**A**-**C**) PAPS injection enhanced locust behavioral mobility and conspecific attraction at 1 h after injection. Behavioral mobility was represented by the total distance moved **(A)** and the duration of time of movement in arena **(B)**. Conspecific attraction was represented by the attraction index **(C)**. **(D)**. PAPS administration promoted locust aggregation at 4 h after injection (*P* < 0.001). Arrows indicated median *P*_greg_ values. N denoted the number of nymphs measured for behavior. (**E-G**) PAPS injection didn’t alter the total distance moved **(E)**, but increased the duration of time of movement in arena (**F**), and altered the attraction index (**G**). The data were shown as mean ± SEM. Asterisks indicate the significance level: *, *P* < 0.05; **, *P* < 0.01; ***, *P* < 0.001.


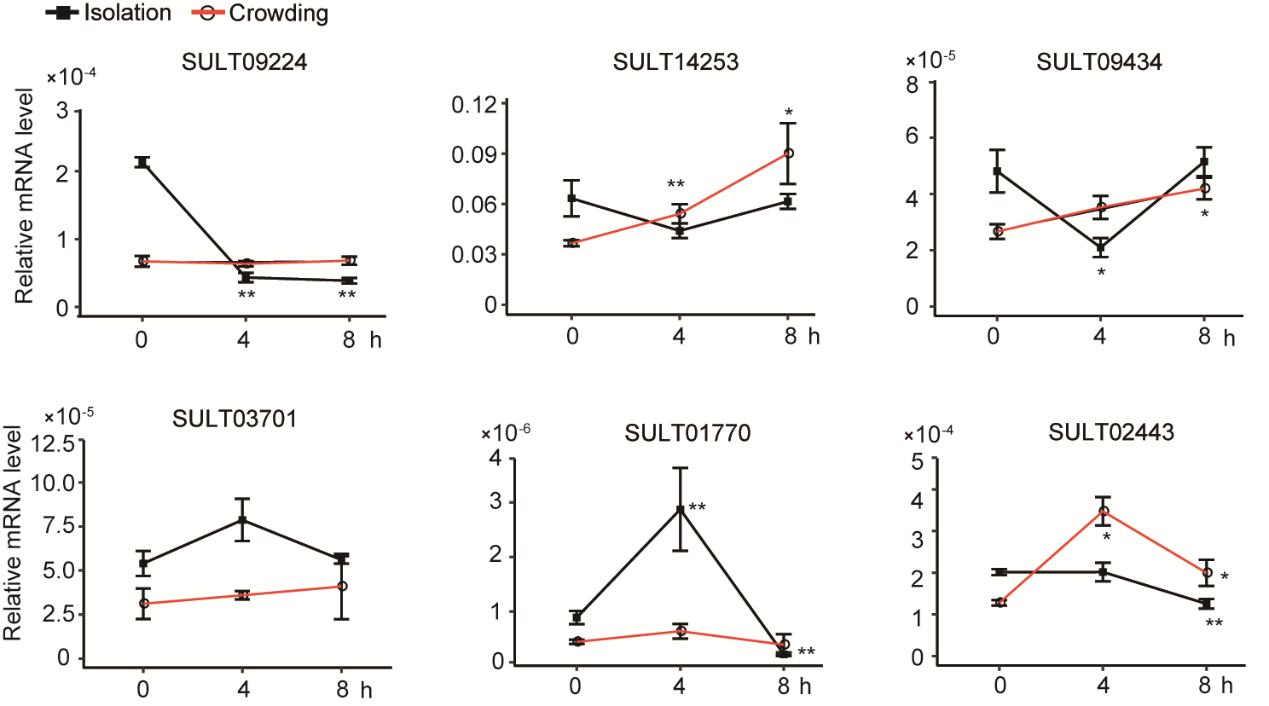


# **Fig. S4. Expression of cytosolic SULT genes in the time course of locust isolation and crowding**. The mRNA level was quantified by qPCR and normalized against that of the internal control *RP49*. The data were shown as mean ± SEM. Asterisks indicate significance differences between each time point and 0 h (i.e., typical G or S locusts): *, *P* < 0.05; **, *P* < 0.01; ***, *P* < 0.001.


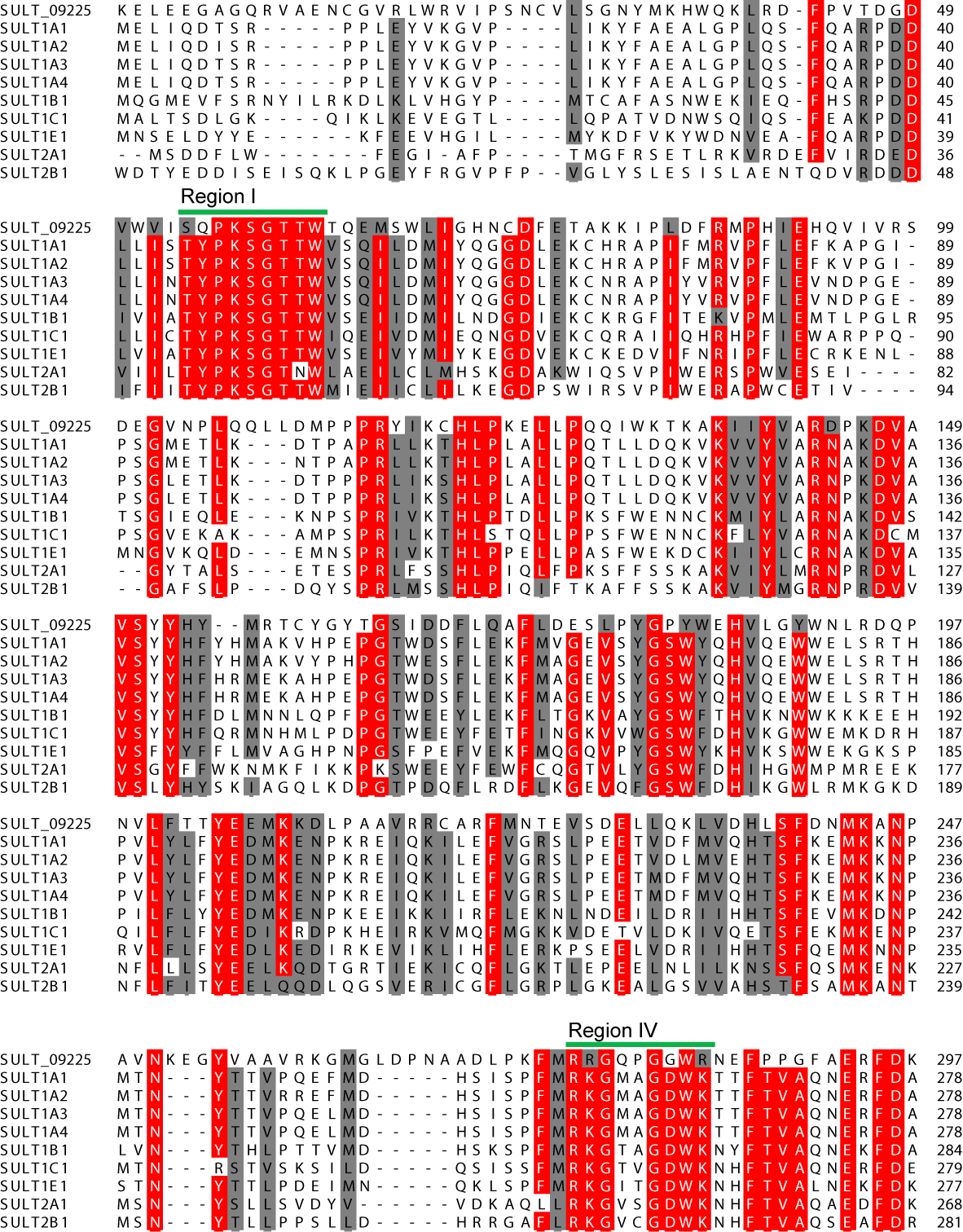


# **Fig. S5.** **Multiple alignment of the locust sulfotransferase SULT09225 with human cytosolic SULTs**. The sequences aligned are part of these SULT proteins. The PAPS-binding regions Region I (PKS/GGTTW/A) and Region IV (GxxGxxK) are indicated by blue line above the sequence. The catalytic residue for the substrate recognition is 252Glu (E). The conserved residues are shaded in red (>=90% identical) and grey (>= 90% similar). See *Materials and Methods* for GenBank accession numbers of these SULTs.


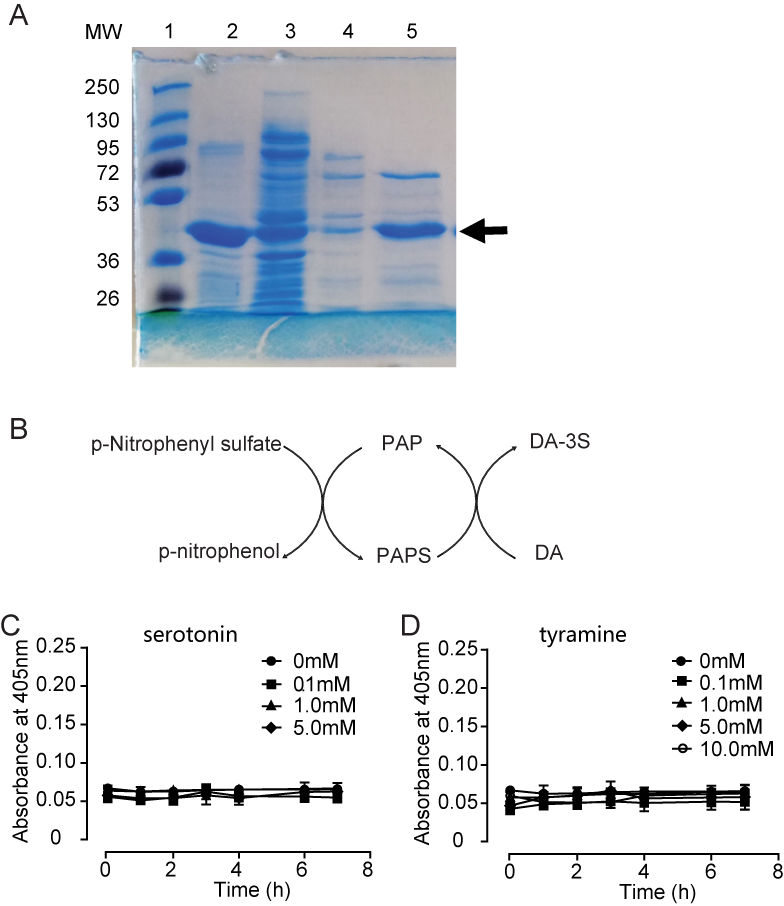


# **Fig. S6. Recombinant expression of SULT09225 and bioamine sulfation catalyzed by SULT09225.** **(A)** The recombinantly expressed SULT09225. 1, marker; 2, the precipitated protein; 3, the supernatant; 4, 50mM protein; 5, 100mM protein. MW, molecular weight (kilodalton). **(B)** Colorimetric phenotyping assay for SULT09225. The substrate in this assay is dopamine (DA). (C, D) SULT09225 did not catalyze the sulfation of serotonin **(C)** and tyramine **(D)**.


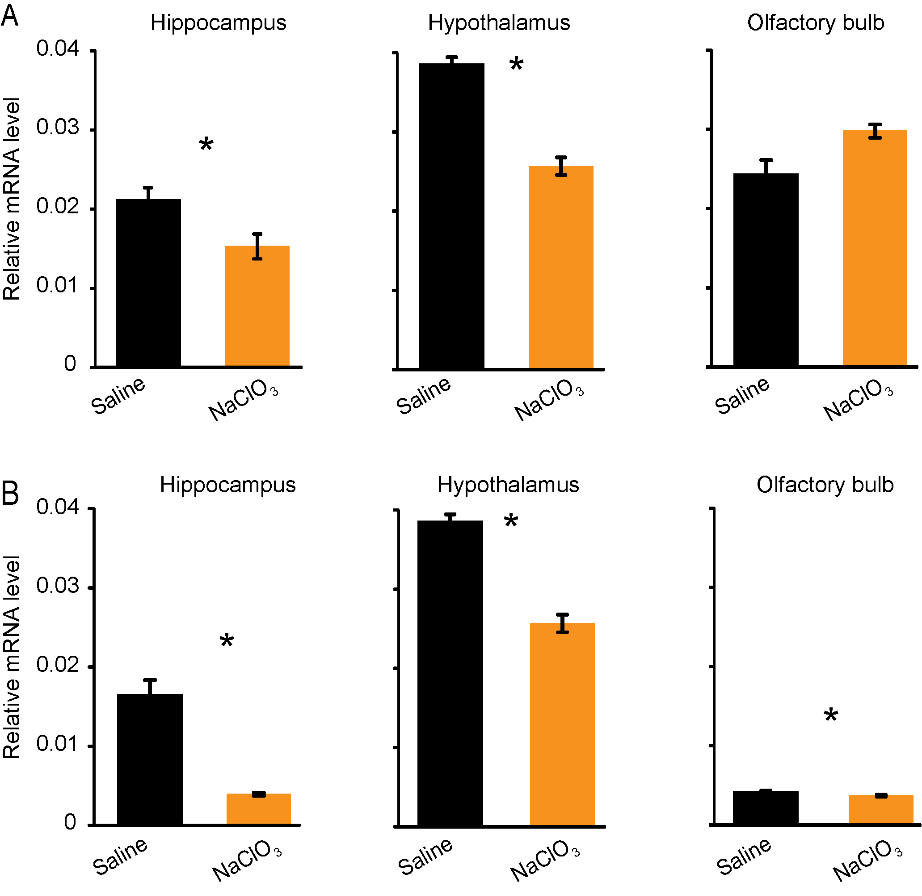


# **Fig. S7.** **NaClO_3_ infusion inhibits *PAPSS* expression in mouse brain.** Effect of NaClO_3_ administration on expression of *PAPSS1* **(A)** or *PAPSS2* **(B)** was examined in hippocampus, hypothalamus and olfactory bulb of brain. Mouse brain was infused with 4 µL 1 M NaClO_3_ or 0.9% NaCl saline solution (i.e., control). Tissues were collected at 60 min after pharmacological infusion. N = 10/group. The mRNA level was quantified by qPCR and normalized against that of the internal control *GAPDH*. NaClO_3_ significantly reduced expression of *PAPSS1* in hippocampus (Students’ *t* test, *P* = 0.013, n = 6) and hypothalamus (*P* < 0.001, n = 6). The NaClO_3_ injection also significantly inhibited expression of *PAPSS2* in hippocampus (*P* = 0.002, n = 6), hypothalamus (*P* = 0.025, n = 6) and olfactory bulb (*P* = 0.049, n = 6). The data were shown as mean ± SEM. Asterisks indicated the significance level of difference evaluated by Student’s *t* test: *, *P* < 0.05.**Table S1. Brain contents of the nine compounds and their parameters used for LC-MS/MS.**

| Monoamine | G brain (ng/brain) | S brain (ng/brain) | Retention time (min) | *m*/*z* in the product ion spectrum, fragmentor, and collision energy |
| --- | --- | --- | --- | --- |
| DA | 1.709 | 1.437 | 29.1 | 154.1→137.1, 65 V, 7 V |
| DA-3S | 0.091 | 0.0696 | 17.4 | 232.0→152.1, 110 V, 15 V |
| DA-4S | - | - | 20.8 | 232.0→152.1, 110V, 15 V |
| TA | 0.083 | 0.068 | 23.4 | 138.1→121.1, 65 V, 7 V |
| TA-4S | - | - | 19.4 | 216.0→136.1, 110 V, 18 V |
| 5-HT | 1.287 | 0.997 | 26.3 | 177.1→160.1, 65 V, 7 V |
| 5-HT-S | - | - | 24.2 | 255.0→175.1, 130 V, 15 V |

Abbreviation**:** DA: dopamine; DA-3S: dopamine-*O*-3-sulfate; DA-4S: dopamine-*O*-4-sulfate; TA: tyramine; TA-4S: tyramine-4-*O*-sulfate; 5-HT: 5-serotonin; 5-HT-S: 5-serotonin-sulfate; G, gregarious phase; S, solitarious phase. – means not detectable.

# **Table S2. Expression of ST genes in the locust brains.** The mRNA level was quantified by qPCR and normalized against that of the internal control gene *rp49*. Each tissue had five biological replicates of ten brains.

| *ST* genes | ds*GFP* | ds*PAPSS* | FC  (ds*PAPSS*/ds*GFP*) | *P* value | G | S | FC  (S/G) | *P* value |
| --- | --- | --- | --- | --- | --- | --- | --- | --- |
| *SULT09224* | 8.5E-05 | 5.2E-05 | -0.39 | ** | 1.9E-05 | 5.7E-05 | 2.09 |  |
| *SULT09225* | 7.0E-05 | 2.1E-05 | -0.71 | ** | 3.7E-04 | 2.7E-04 | -0.26 | * |
| *SULT14253* | 1.6E-02 | 1.6E-02 | -0.02 |  | 4.0E-02 | 2.1E-02 | -0.48 | ** |
| *SULT09434* | 5.8E-04 | 3.8E-05 | -0.93 | ** | 5.3E-05 | 1.3E-05 | -0.76 | ** |
| *SULT03701* | 4.8E-04 | 2.4E-04 | -0.49 | ** | 2.8E-04 | 6.6E-05 | -0.77 | ** |
| *SULT01770* | 8.8E-06 | 2.2E-06 | -0.75 | ** | 1.0E-03 | 5.7E-04 | -0.42 | ** |
| *SULT02443* | 3.0E-04 | 6.4E-04 | 1.13 | ** | 6.6E-04 | 3.8E-04 | -0.43 | ** |
| *HS6ST1* | 5.0E-02 | 5.2E-02 | 0.04 |  | 1.2E-01 | 1.0E-01 | -0.10 |  |
| *HS2ST1* | 1.6E-02 | 1.7E-02 | 0.09 |  | 6.7E-02 | 4.6E-02 | -0.31 | ** |
| *HS2ST/pipe* | 1.2E-03 | 1.6E-03 | 0.38 | ** | 1.4E-03 | 4.8E-03 | 2.49 | * |
| *TPST2* | 1.7E-03 | 1.6E-03 | -0.03 |  | 2.8E-03 | 2.1E-03 | -0.25 |  |
| *NDST4* | 3.4E-02 | 4.3E-02 | 0.26 | * | 4.1E-02 | 2.8E-02 | -0.32 | * |
| *CHST5* | 4.3E-04 | 7.6E-04 | 0.77 |  | 6.3E-05 | 3.2E-05 | -0.49 |  |
| *CHST11* | 9.4E-02 | 1.7E-01 | 0.85 | ** | 1.8E-01 | 1.1E-01 | -0.37 | ** |
| *CHST10* | 5.1E-02 | 8.1E-02 | 0.58 | * | 1.6E-01 | 1.1E-01 | -0.34 | ** |
| *CHST8* | 5.7E-03 | 7.6E-03 | 0.33 | ** | 3.8E-03 | 6.4E-03 | 0.68 |  |

*P* value from students’ t test: *, *P* < 0.05; **, *P* < 0.01; ****, P* < 0.001.

FC represents fold change.

# **Table S3. Sequences of all primers used in the experiments**

| Primer name | Gene  (GenBank Accession number) | Sequence | Length (bp) | Note |
| --- | --- | --- | --- | --- |
| *L. migratoria* | | | | |
| PapssF1 | *PAPSS* (KT000398) | CATCACATAGAGGACACCCTTACA | 135 | For quantitative PCR |
| PapssR1 |  | GCTCAAGTGGGGTTAGACGATA |  |  |
| dsPapssF | *PAPSS* (KT000398) | TCTTTGAACCTCAGCGTAA | 771 | For RNAi and RACE |
| dsPapssR |  | TTCCCATCAGCAGTAGAGT |  |  |
| PapssF2 | *PAPSS* (KT000398) | GTGTCTGCCGTCAGTTTG | 333 | For *In Situ* hybryidization |
| PapssR2 |  | CATCCTTCGTCCAACCAC |  |  |
| PapssF3 | *PAPSS* (KT000398) | GCCAGATACTCGGCAACC | 336 |  |
| PapssR3 |  | ATGGCAAATCACTTTCCTTG |  |  |
| SULT_09225F | *SULT_09225* (KT000399) | TGCTGGGCTACTGGAATCTC | 204 | For quantitative PCR |
| SULT_09225R |  | CATAGCCCTCCTTGTTGACC |  |  |
| dsSULT_9225F | *SULT_09225* (KT000399) | GGGTGGCGGAGAACTGCGG | 317 | For dsRNA synthesis |
| dsSULT_9225R |  | GCGGGGGCGGCATGTCTAG |  |  |
| SULT09225F | *SULT09225* (KT000399) | ATGGATCTGGAGTTCAAGGAG | 966 | For amplifying coding sequence |
| SULT09225R |  | CTAGGCGTGTCCCCCGGAG |  |  |
| SULT_09434F | *SULT_09434* (KT000402) | TCCCCGACTGCCTGCTGG | 428 | For quantitative RT-PCR |
| SULT_09434R |  | CTGCTGCATAGTTTCTTTAGC |  |  |
| SULT_14253F | *SULT_14253* (KT000401) | TACAGCGGCAACATCAAGC | 279 |  |
| SULT_09434R |  | GAGAATGTTTGGTTCGTGGC |  |  |
| SULT_09224F | *SULT_09224* (KT000400) | CAAACTGACGCCTCCACG | 317 |  |
| SULT_09224R |  | GCTGTTTCATCTGCTCGTC |  |  |
| SULT_03195F | *TPST2* (KT000409) | GGCGTGCCCCGTTCCGGC | 163 |  |
| SULT_03195R |  | CCACGTCCTCCGGGATGTGG |  |  |
| SULT_12816F | *HS2ST/pipe* (KT000408) | CTGCCGGTAGCTCGACAG | 338 |  |
| SULT_12816R |  | CTTCTCATTGATGTCCGGGG |  |  |
| SULT_03945F | *HS2ST1* (KT000407) | CTGATTCGGAAACCACTG | 234 |  |
| SULT_03945R |  | TTCTTCAAGTGCCCAGTC |  |  |
| SULT_05945F | *HS6ST1* (KT000406) | TTTGGCTTTGGTGGGTTGC | 390 |  |
| SULT_05945R |  | CTCTGTCTCCCAGGCAAT |  |  |
| SULT_03983F | *NDST4* (KT000410) | ACTCCCTCAGGCTAAACTC | 429 |  |
| SULT_03983R |  | TGCCTCCCTTTGCTACGC |  |  |
| SULT_12197F | *CHST5* (KT000411) | GACTGGCTCGGGATACAC | 482 |  |
| SULT_12197R |  | CTGGTAGCCCTCCATCTC |  |  |
| SULT_01770F | *SULT_0177*  (KT000404) | TTATCATCTATGTGGCAAGG | 155 |  |
| SULT_01770R |  | GAACATTCGTCCAGTAGG |  |  |
| SULT_07706F | *CHST8* (KT000414) | GAGTGAAATAGCCAGGGAAGC | 357 |  |
| SULT_07706R |  | CGCTGAGAAAGGTCGGTGTC |  |  |
| SULT_00767F | *CHST10* (KT000413) | TTTGCTTTCTGCCTACCG | 331 |  |
| SULT_00767R |  | AAAGTGGGAGGGTAAGAG |  |  |
| SULT_08914F | *CHST11* (KT000412) | ATCCGTGTCCTTCCTTATTG | 341 |  |
| SULT_08914R |  | GGTCTTCCTCAAGGGTTTC |  |  |
| SULT_02443F | *SULT_02443* (KT000405) | TGGCGAGGAGCCCCAAGG | 294 |  |
| SULT_02443R |  | GCCTCGACGCTTGGACCAC |  |  |
| SULT_03701F | *SULT_03701* (KT000403) | ATGGGTGGTCACATTTCCTAG | 369 |  |
| SULT_03701R |  | GACTGATTAAGAAGCTCCTGC |  |  |
| *Mus musculus* | | | | |
| PAPSS1F | *PAPSS1* (NM_011863.2) | GCTGTACTGGAGGAAAGGGT | 178 | For quantitative RT-PCR |
| PAPSS1R |  | TTGTCTCAGGATGGGGCATT |  |  |
| PAPSS2F | *PAPSS2* (NM_011864.3) | CTGGACCGGATTTACTGG | 264 |  |
| PAPSS2R |  | CTGCTTCATACGCCACATC |  |  |
| GAPDH_F | *GAPDH* (NM_008084.3) | GGAGAAACCTGCCAAGTATG | 117 |  |
| GAPDH_R |  | CTGTTGAAGTCGCAGGAGAC |  |  |
